# Rice transcription factor bHLH25 confers resistance to multiple diseases by sensing H_2_O_2_

**DOI:** 10.1038/s41422-024-01058-4

**Published:** 2025-01-14

**Authors:** Haicheng Liao, Yu Fang, Junjie Yin, Min He, Yingjie Wei, Juan Zhang, Shuang Yong, Jiankui Cha, Li Song, Xiaobo Zhu, Xixi Chen, Ján Kováč, Qingqing Hou, Zhaotang Ma, Xiaogang Zhou, Lin Chen, Emi Yumoto, Tian Yang, Qi He, Wei Li, Yixin Deng, Haoxuan Li, Mingwu Li, Hai Qing, Lijuan Zou, Yu Bi, Jiali Liu, Yihua Yang, Daihua Ye, Qi Tao, Long Wang, Qing Xiong, Xiang Lu, Yongyan Tang, Ting Li, Bingtian Ma, Peng Qin, Yan Li, Wenming Wang, Yangwen Qian, Jaroslav Ďurkovič, Koji Miyamoto, Mawsheng Chern, Shigui Li, Weitao Li, Jing Wang, Xuewei Chen

**Affiliations:** 1 State Key Laboratory of Crop Gene Exploration and Utilization in Southwest China, Sichuan Agricultural University, Chengdu, Sichuan China; 2 Department of Phytology, Technical University in Zvolen, Zvolen, Slovakia; 3 Advanced Instrumental Analysis Center, Teikyo University, Utsunomiya, Tochigi Japan; 4 Rice Research Institute, Sichuan Agricultural University, Chengdu, Sichuan China; 5 WIMI Biotechnology Company Limited, Sanya, Hainan China; 6 Department of Biosciences, Faculty of Science and Engineering, Teikyo University, Utsunomiya, Tochigi Japan; 7 Department of Plant Pathology, University of California, Davis, CA USA

**Keywords:** Plant immunity, Plant molecular biology

## Abstract

Hydrogen peroxide (H_2_O_2_) is a ubiquitous signal regulating many biological processes, including innate immunity, in all eukaryotes. However, it remains largely unknown that how transcription factors directly sense H_2_O_2_ in eukaryotes. Here, we report that rice basic/helix-loop-helix transcription factor bHLH25 directly senses H_2_O_2_ to confer resistance to multiple diseases caused by fungi or bacteria. Upon pathogen attack, rice plants increase the production of H_2_O_2_, which directly oxidizes bHLH25 at methionine 256 in the nucleus. Oxidized bHLH25 represses *miR397b* expression to activate lignin biosynthesis for plant cell wall reinforcement, preventing pathogens from penetrating plant cells. Lignin biosynthesis consumes H_2_O_2_ causing accumulation of non-oxidized bHLH25. Non-oxidized bHLH25 switches to promote the expression of *Copalyl Diphosphate Synthase 2* (*CPS2*), which increases phytoalexin biosynthesis to inhibit expansion of pathogens that escape into plants. This oxidization/non-oxidation status change of bHLH25 allows plants to maintain H_2_O_2_, lignin and phytoalexin at optimized levels to effectively fight against pathogens and prevents these three molecules from over-accumulation that harms plants. Thus, our discovery reveals a novel mechanism by which a single protein promotes two independent defense pathways against pathogens. Importantly, the bHLH25 orthologues from available plant genomes all contain a conserved M256-like methionine suggesting the broad existence of this mechanism in the plant kingdom. Moreover, this Met-oxidation mechanism may also be employed by other eukaryotic transcription factors to sense H_2_O_2_ to change functions.

## Introduction

Hydrogen peroxide (H_2_O_2_), a main reactive oxygen species (ROS), plays central roles in many biological processes in eukaryotes.^1–3^ Plants respond to pathogen infection with a burst of oxidants including superoxide anions (O_2_^–^) in the apoplastic spaces produced by nicotinamide adenine dinucleotide phosphate (NADPH) oxidases to inhibit pathogen growth.^4–6^ These extracellular oxidants are converted by peroxidases to H_2_O_2_ as the final product, which is transported into the host cell to trigger immune responses.^3,7,8^ The role of H_2_O_2_ in immunity was first reported in 1974 for animals^9^ and in 1983 for plants.^10^ Cells sense H_2_O_2_ and transduce the signal mainly through H_2_O_2_-mediated oxidative post-translational modifications of sulfur-containing amino acids cysteine and methionine in proteins at different subcellular locations.^3,11,12^

It has taken over four decades to find proteins other than peroxidases that directly sense H_2_O_2_ to control innate immunity. Master immune regulator non-expressor of pathogenesis-related 1 (NPR1) was recently reported to function as a sensor of redox rhythm in *Arabidopsis* to gate plant immune response towards the morning and minimize costs on growth at night.^13^ Redox sensor Quiescin sulfhydryl oxidase homolog 1 (QSOX1) regulates ROS levels in *Arabidopsis* to modulate plant immunity.^14^ However, whether NPR1 and QSOX1 directly sense and become oxidized by H_2_O_2_ remains undetermined. Interestingly, leucine-rich-repeat receptor kinase hydrogen-peroxide-induced Ca^2+^ increases 1 (HPCA1) protein directly recognizes H_2_O_2_ in *Arabidopsis*,^15^ but whether HPCA1 regulates innate immunity is undetermined. However, our current knowledge about transcription factors which directly sense H_2_O_2_ in the nucleus and trigger global gene expression remains quite limited. It is worth noticing that H_2_O_2_ can be transported into the nucleus to regulate immune responses.^16^ Thus, a clear gap remains in the identification of transcription factors that directly sense H_2_O_2_ to regulate immunity and in the understanding of their underlying mechanisms.

## Results

### H_2_O_2_ promotes immunity partially through *OsLAC7/28/29-*mediated lignin biosynthesis

**Fig. 1:**
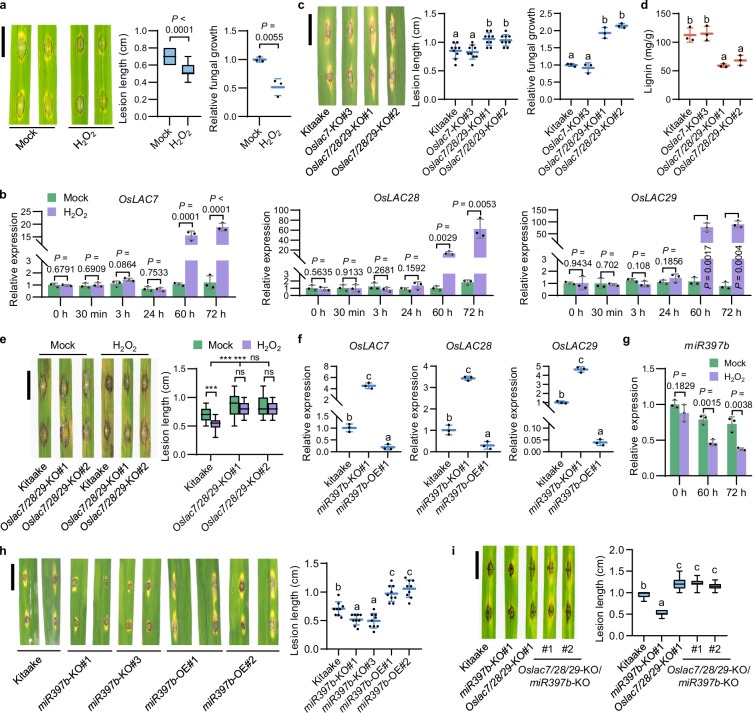
H_2_O_2_ promotes *OsLAC7/28/29*-mediated lignin biosynthesis and disease resistance by repressing *miR397b* expression. **a** Three-week-old Kitaake plants were pre-treated with or without 1 mM H_2_O_2_ on roots for 72 h, then their leaves were inoculated with *M. oryzae* Zhong10-8-14. Lesion length (*n* ≥ 18 lesions) and fungal growth (*n* = 3 technical replicates) at 7 days post inoculation (dpi) are shown. Mock treatment indicates treatment without H_2_O_2_. **b** *OsLAC7/28/29* expression levels in three-week-old Kitaake leaves at 0–72 hours post treatment (hpt) with or without 1 mM H_2_O_2_ on roots (*n* = 3 technical replicates). **c** Lesion length (*n* = 9 lesions) and fungal growth (*n* = 3 technical replicates) of three-week-old Kitaake, *Oslac7*-KO and *Oslac7/28/29*-KO plants at 7 dpi with Zhong10-8-14. **d** Lignin contents in four-week-old Kitaake, *Oslac7*-KO and *Oslac7/28/29*-KO plants (*n* = 3 biological replicates). **e** Three-week-old triple KO of *OsLAC7/28/29* and Kitaake plants were pre-treated with or without 1 mM H_2_O_2_ on roots for 72 h, then their leaves were inoculated with Zhong10-8-14. Lesion lengths (means ± SD, *n* = 30 lesions) at 7 dpi are shown. **f** *OsLAC7/28/29* RNA levels in three-week-old Kitaake, *miR397b*-KO and *miR397b*-OE plants (*n* = 3 technical replicates). **g** *miR397b* levels in three-week-old Kitaake 0, 60 and 72 hpt with or without 1 mM H_2_O_2_ on roots (*n* = 3 technical replicates). **h** Lesion length (*n* = 9 lesions) of three-week-old Kitaake, *miR397b*-KO and *miR397b*-OE plants at 7 dpi with Zhong10-8-14. **i** Lesion lengths (*n* = 20 lesions) of three-week-old Kitaake, *miR397b*-KO, *Oslac7/28/29*-KO and double mutant *Oslac7/28/29*-KO/*miR397b*-KO plants at 7 dpi with Zhong10-8-14. Data are means ± SD and analyzed by two-tailed Student’s *t*-test (**a**, **b**, **g**), one-way ANOVA with Least Significant Difference (LSD) test (**c**, **d**, **f**, **h**, **i**), and two-way ANOVA with Tukey’s test at ***P < 0.001; ns, not significant (**e**). Scale bar, 1 cm (**a**, **c**, **e**, **h**, **i**). Experiments were done with three biologically independent replications.

We treated rice plants with exogenous H_2_O_2_ on roots and detected the accumulation of H_2_O_2_ content and *OsPR1b* and *OsPR10b* mRNAs in rice leaves (Supplementary information, Fig. S1a–d). Plants treated with H_2_O_2_ showed enhanced resistance to *Magnaporthe oryzae*, the causal pathogen of the destructive blast disease (Fig. 1a), confirming that H_2_O_2_ promotes plant immunity. We then performed global transcriptomic analysis via RNA-sequencing and identified 1596 genes up-regulated by H_2_O_2_ treatment (Supplementary information, Fig. S1e and Table S1). Gene ontology analysis revealed that these up-regulated genes fall into different biological processes (Supplementary information, Fig. S1f and Table S2), with carbohydrate metabolism, response to oxidative stress, and cell wall biogenesis as the most significantly induced processes (Supplementary information, Fig. S1f).

Cell wall is the first physical barrier that plants use to defend against pathogen penetration at early infection.^17^ We therefore screened for most highly up-regulated genes related to cell wall biogenesis and found five genes involved in pectin biosynthesis, five in xyloglucan biosynthesis, one in cellulose biosynthesis, and three in lignin biosynthesis (Supplementary information, Table S2). Because we found lignin accumulation after H_2_O_2_ treatment (Supplementary information, Fig. S1g) and lignin is a key molecule that strengthens cell wall to enhance disease resistance,^18,19^ we focused on the three lignin biosynthesis genes, *OsLAC7, OsLAC28* and *OsLAC29* (abbreviated as *OsLAC7/28/29*), which encode laccases that polymerize monolignols to form lignin polymers for cell wall reinforcement.^20,21^ We then validated the induction of *OsLAC7/28/29* expression by H_2_O_2_ in rice by reverse transcription quantitative PCR (RT-qPCR) (Fig. 1b).

We then determined the role of *OsLAC7/28/29* in plant disease resistance. Single knockout (KO) plants for any of *OsLAC7/28/29* and double KO plants of *OsLAC7/29* showed no changes in disease resistance or lignin contents (Supplementary information, Figs. S2, S3 and S4a–e). Most importantly, triple KO plants of *OsLAC7/28/29* showed decreased disease resistance and lower lignin contents (Fig. 1c, d; Supplementary information, Fig. S4f–k). Plants overexpressing anyone of *OsLAC7/28/29* accumulated more lignin contents and stronger disease resistance than Kitaake plants (Supplementary information, Figs. S4c–e, l–n). Besides, overexpression plants displayed thicker sclerenchyma cells and cell walls than Kitaake plants (Supplementary information, Fig. S4o, p). These results suggest that *OsLAC7/28/29* positively regulate lignin biosynthesis and disease resistance in a functionally redundant manner. More importantly, H_2_O_2_-induced disease resistance was significantly weakened in *Oslac7/28/29* triple KO plants compared to the wild-type Kitaake plants (Fig. 1e), indicating that H_2_O_2_ induces disease resistance partially through *OsLAC7/28/29*.

### *OsLAC7/28/29* are targeted and repressed by *miR397b*

We next examined *OsLAC7/28/29* promoters for response to H_2_O_2_. Although *OsLAC7/28/29* promoters are enriched with *cis*-elements for TATA-box-binding proteins (TBPs) and CAAT-box-binding factors (CBFs) (Supplementary information, Fig. S5a and Table S3), neither rice *TBP* nor *CBF* genes were induced or repressed by H_2_O_2_ (Supplementary information, Fig. S5b, c and Table S4). We then cloned 18 TBPs and CBFs and performed transactivation assays to determine their transcriptional regulatory activity on *OsLAC7/28/29* promoters. The results showed that none of these 18 TBP or CBF transcription factors simultaneously activated *OsLAC7/28/29* promoters (Supplementary information, Table S5), indicating that some factor(s) other than TBPs and CBFs promote *OsLAC7/28/29* expression upon H_2_O_2_ induction. As microRNAs (miRNAs) are well-known for regulating gene expression,^22^ we screened for potential miRNA-binding sites (MBS) and found that only one *miR397b*-binding site was commonly shared by *OsLAC7/28/29* (Supplementary information, Fig. S5d, e and Table S6). Interestingly, among all genes induced by H_2_O_2_, only *OsLAC7/28/29* mRNAs contain *miR397b* MBS (Supplementary information, Table S6). These results suggest that *miR397b* may specifically target *OsLAC7/28/29*.

In transactivation assays, *Nicotiana benthamiana* leaves co-expressing *35S:miR397b* and *35S:MBS*^*LACs*^*-YFP* (yellow fluorescent protein sequence tagged with *miR397b* MBS) showed lower *YFP* RNA levels than those co-expressing *35S:miR397b* and *35S:mMBS*^*LACs*^*-YFP* (YFP fused with mutated MBS abolishing *miR397b* recognition) (Supplementary information, Fig. S5f, g). Nevertheless, *35S:MBS*^*LACs*^*-YFP* alone and *35S:mMBS*^*LACs*^*-YFP* alone expressed similar *YFP* RNA levels (Supplementary information, Fig. S5g). RT-qPCR assays showed that *miR397b-*KO plants expressed higher *OsLAC7/28/29* levels, whereas *miR397b* overexpression (*miR397b-*OE) plants expressed lower *OsLAC7/28/29* levels than Kitaake plants (Fig. 1f; Supplementary information, Fig. S5h–j). In addition, we found that H_2_O_2_ represses *miR397b* expression (Fig. 1g) and that *miR397b*-KO blocks the induction of *OsLAC7*/*28*/*29* expression by H_2_O_2_ (Supplementary information, Fig. S6a). These results indicate that H_2_O_2_ represses *miR397b* expression to increase *OsLAC7/28/29* expression.

When inoculated with *M. oryzae*, *miR397b-*KO plants developed smaller lesions and harbored less *M. oryzae* than Kitaake plants, whereas *miR397b*-OE plants responded oppositely (Fig. 1h; Supplementary information, Fig. S6b, c). *miR397b*-KO plants displayed thicker sclerenchyma cell walls with more lignin; *miR397b*-OE plants showed the opposite (Supplementary information, Fig. S6d–f). These results suggest that the *miR397b*-laccases module regulates disease resistance by controlling lignin accumulation and cell wall thickness. Additionally, *Oslac7/28/29*-KO/*miR397b*-KO plants and *Oslac7/28/29*-KO plants displayed the same levels of blast resistance, both lower than the wild-type Kitaake and *miR397b*-KO plants (Fig. 1i), indicating that the effects of *miR397b* in disease resistance completely rely on the suppression of *OsLAC7/28/29*.

### bHLH25 represses *miR397b* to enhance lignin biosynthesis and disease resistance

**Fig. 2:**
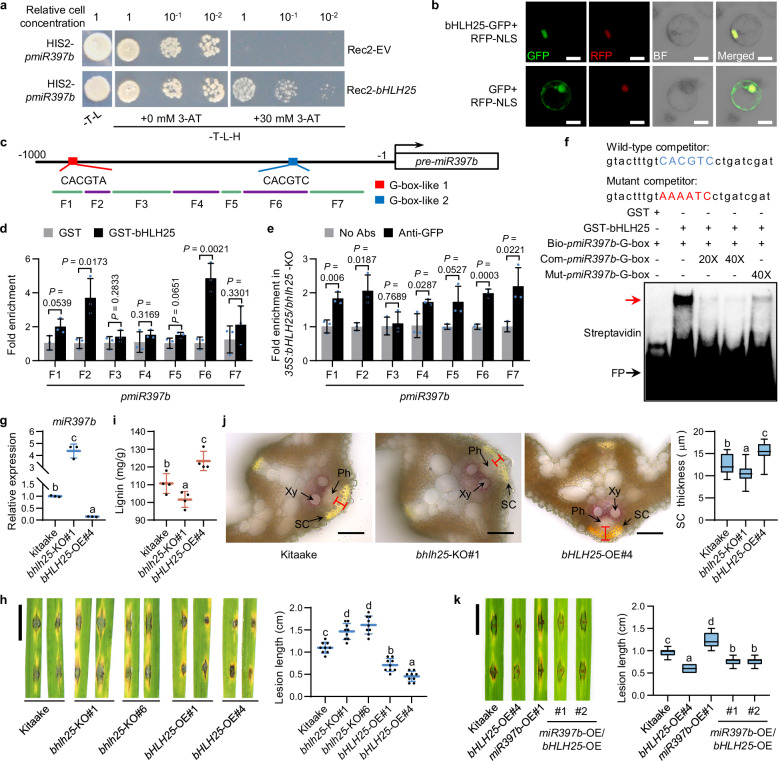
bHLH25 represses *miR397b* expression to enhance lignin biosynthesis and disease resistance. **a** Yeast one-hybrid assay for binding of bHLH25 to *pmiR397b*. 3-amino-1,2,4-triazole (3-AT) was used to inhibit leaky reporter expression. **b** Subcellular localization of bHLH25 in rice protoplasts. RFP-NLS, red fluorescence protein with a nuclear localization signal. **c** The G-box-like motifs and seven fragments of *pmiR397b* are shown. **d** Fold enrichment of *pmiR397b* in fragmented rice DNA pulled down by GST-bHLH25 in DAP-qPCR assay (*n* = 3 technical replicates). GST was used as a negative control. **e** Fold enrichment of fragmented DNA of *pmiR397b* pulled down by bHLH25-YFP in ChIP-qPCR assay (*n* = 3 technical replicates). bHLH25-YFP was immunoprecipitated from plants overexpressing *bHLH25-YFP* in *bhlh25*-KO background by protein A-magnetic beads coupled with anti-GFP or without antibodies (No Abs, negative control). **f** EMSA for examining the binding of bHLH25 to *pmiR397b*. GST-bHLH25 and GST (negative control) were incubated with biotin-labeled probe (bio-*pmiR397b*-G-box) and unlabeled competitors containing wild-type G-box-like-2 motif (in blue) of *pmiR397b* (com-*pmiR397b*-G-box) or mutant motif (in red, mut-*pmiR397b*-G-box). The red arrow indicates biotin-labeled probes bound to GST-bHLH25. FP, free probes. **g** *miR397b* levels (*n* = 3 technical replicates) in three-week-old Kitaake, *bhlh25*-KO and *bHLH25*-OE plants. **h** Lesion length (*n* = 9 lesions) of 3-week-old Kitaake, *bhlh25*-KO and *bHLH25*-OE plants at 7 dpi with Zhong10-8-14. **i** Lignin contents (*n* = 4 biological replicates) in three-week-old Kitaake, *bhlh25*-KO and *bHLH25*-OE plants. **j** Histochemical staining of cross-sectioned leaves with phloroglucinol-HCl and thickness of sclerenchyma cells in three-week-old Kitaake, *bhlh25*-KO and *bHLH25*-OE plants (*n* = 21 cells). **k** Lesion length (*n* = 20 lesions) of three-week-old Kitaake, *bHLH25*-OE, *miR397b*-OE, and double mutant *miR397b*-OE/*bHLH25*-OE plants at 7 dpi with Zhong10-8-14. Data are means ± SD and analyzed by two-tailed Student’s *t*-test (**d**, **e**) and one-way ANOVA with LSD test (**g**–**k**). Scale bars, 10 μm (**b**), 50 μm (**j**) and 1 cm (**h**, **k**). Experiments were done with three biologically independent replications.

To search for transcription factors that regulate *miR397b* expression in response to H_2_O_2_, we identified various *cis*-elements in *miR397b* promoter (*pmiR397b*) (Supplementary information, Table S3) which makes it difficult to identify relevant transcription factors. Therefore, we used yeast one-hybrid to screen a rice cDNA library with *pmiR397b* as the bait and identified a basic/helix-loop-helix transcription factor, bHLH25, which is localized in rice nucleus and able to bind to *pmiR397b* in yeast (Fig. 2a, b). DNA affinity purification coupled with quantitative PCR (DAP-qPCR) and chromatin immunoprecipitation coupled with qPCR (ChIP-qPCR) using *bhlh25*-KO plants overexpressing *bHLH25* showed that bHLH25 is enriched in rice chromatin containing *pmiR397b* with a G-box-like-2 motif (Fig. 2c–e; Supplementary information, Fig. S7a). Electrophoretic mobility shift assay (EMSA) showed that bHLH25 directly bound to biotin-labeled G-box-like-2 motif in *pmiR397b* (bio-*pmiR397b*-G-box), and un-labeled competitors carrying wild-type G-box-like-2 motif (com-*pmiR397b*-G-box) markedly reduced the binding of bHLH25 to G-box-like-2 motif, whereas competitors with mutant G-box-like-2 motif (mut-*pmiR397b*-G-box) carried significantly reduced competing ability (Fig. 2f), indicating that bHLH25 binds to *pmiR397b* mainly through the G-box-like-2 motif, but also partially through nucleotides flanking the G-box-like-2 motif. These results are also consistent with previous reports showing that nucleotides flanking the core *cis*-elements on promoters are also important for the binding specificity and affinity of bHLH-type transcription factors.^23,24^

We further performed a transactivation assay and found that *bHLH25* overexpression (*bHLH25*-OE) inhibited the RNA level of *YFP* driven by *pmiR397b* in *N. benthamiana* (Supplementary information, Fig. S7b). RT-qPCR assays showed that *miR397b* expression decreased in *bHLH25*-OE rice transgenic plants, but increased in *bhlh25*-KO plants compared with that in Kitaake (Fig. 2g; Supplementary information, Fig. S7c–h). In contrast, *OsLAC7/28/29* mRNA levels increased in *bHLH25*-OE plants but decreased in *bhlh25*-KO plants (Supplementary information, Fig. S7i, j). Moreover, we found that *bHLH25* KO abolished the H_2_O_2_-mediated repression of *miR397b* expression (Supplementary information, Fig. S7k). These results suggest that the repression of *miR397b* expression by H_2_O_2_ is dependent on bHLH25.

We next determined whether *bHLH25* regulates disease resistance. *bhlh25-*KO plants exhibited larger lesions and harbored more fungus than Kitaake, whereas *bHLH25*-OE plants responded oppositely (Fig. 2h; Supplementary information, Fig. S7l). In agreement, *bHLH25*-OE plants showed more lignin and thicker sclerenchyma cell walls than Kitaake plants, whereas *bhlh25*-KO plants showed the opposite (Fig. 2i, j; Supplementary information, Fig. S7m). Taken together, these results indicate that *bHLH25* promotes lignin biosynthesis, cell wall reinforcement and disease resistance by repressing *miR397b* expression. In addition, the disease resistance level of *miR397b*-OE/*bHLH25*-OE plants was much higher than that of *miR397b*-OE plants, but significantly lower than that of *bHLH25*-OE (Fig. 2k), suggesting that the bHLH25-conferred disease resistance is partially mediated by *miR397b*.

### bHLH25 promotes *CPS2*-mediated phytoalexin biosynthesis to enhance disease resistance

**Fig. 3:**
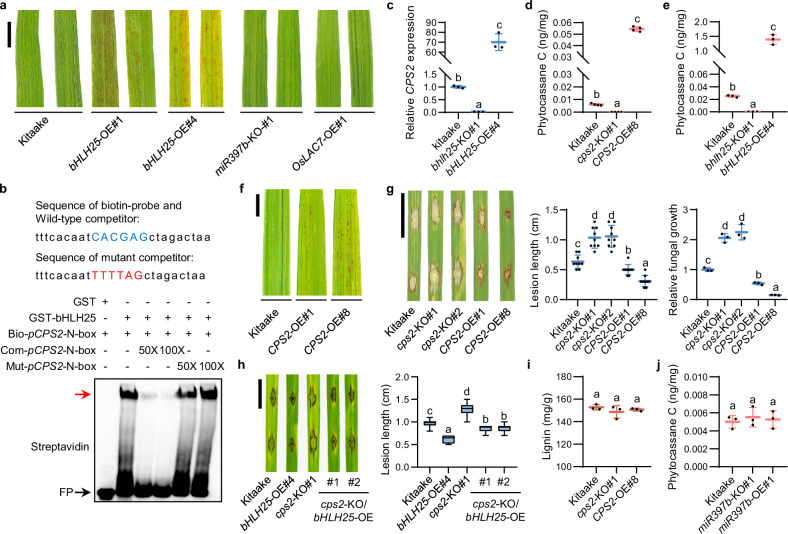
bHLH25 promotes *CPS2*-mediated phytoalexin biosynthesis and disease resistance in rice in a manner independent of *miR397b.* **a** Representative leaves of Kitaake, *bHLH25*-OE, *miR397b*-KO and *OsLAC7*-OE plants at the tillering stage. **b** Binding of bHLH25 to the N-box-like motif in *pCPS2* in EMSA. GST-bHLH25 and GST (negative control) were incubated with biotin-labeled probe (bio-*pCPS2*-N-box) with or without unlabeled wild-type (com-*pCPS2*-N-box) or mutant competitor (mut-*pCPS2*-N-box). Wild-type and mutant N-box-like sequences are highlighted in blue and red, respectively. The red arrow indicates biotin-labeled probes bound to GST-bHLH25. **c** *CPS2* RNA levels in three-week-old Kitaake, *bhlh25*-KO and *bHLH25*-OE plants (*n* = 3 technical replicates). **d** Phytocassane C contents in three-week-old Kitaake, *cps2*-KO and *CPS2*-OE plants (*n* = 3 biological replicates). **e** Phytocassane C contents in three-week-old Kitaake, *bhlh25*-KO and *bHLH25*-OE plants (*n* = 3 biological replicates). **f** Representative leaves of Kitaake and *CPS2*-OE plants at the tillering stage. **g** Lesion length (*n* = 9 lesions) and fungal growth (*n* = 3 technical replicates) of three-week-old Kitaake, *cps2*-KO and *CPS2*-OE plants 7 dpi with Zhong10-8-14. **h** Lesion lengths (*n* = 20 lesions) of three-week-old Kitaake, *bHLH25*-OE, *cps2*-KO, and double mutant *cps2*-KO/*bHLH25*-OE plants at 7 dpi with Zhong10-8-14. **i** Lignin contents in three-week-old Kitaake, *cps2*-KO and *CPS2*-OE plants (*n* = 3 biological replicates). **j** Phytocassane C contents in three-week-old Kitaake, *miR397b*-KO and *miR397b*-OE plants (*n* = 3 biological replicates). Data are means ± SD and analyzed by one-way ANOVA with LSD test (**d**, **g**, **h**–**j**) or Dunnett’s test (**c**, **e**). Scale bar, 1 cm (**a**, **f**–**h**). Experiments were done with three biologically independent replications.

Interestingly, *bHLH25*-OE plants also exhibited a cell death phenotype on leaves in the absence of pathogen attack (Fig. 3a), which was not observed in either *miR397b*-KO or *OsLAC7*-OE plants. We speculated that *bHLH25* likely regulates other defense pathway(s) independent of the *miR397b*-laccases pathway. A previous study showed that bHLH25 putatively bound to the N-box-like motif in the *CPS2* promoter (*pCPS2*).^25^ CPS2 produces phytocassanes,^26^ a type of antimicrobial phytoalexins, which attracted us because of their ability to directly inhibit *M. oryzae* spore germination and fungal hypha growth and to trigger cell death.^27–29^ Thus, we further examined whether bHLH25 regulates *CPS2* expression.

EMSA validated binding of bHLH25 to the N-box-like motif in *pCPS2* (Fig. 3b). Transactivation assays revealed that *bHLH25* enhanced the RNA level of *YFP* driven by *pCPS2* in *N. benthamiana* (Supplementary information, Fig. S8a). Consistently, *CPS2* expression was elevated in *bHLH25*-OE plants but deeply repressed in *bhlh25*-KO plants (Fig. 3c; Supplementary information, Fig. S8b). Besides, the contents of phytocassane C were higher in *CPS2* overexpression (*CPS2*-OE) and *bHLH25*-OE plants than in Kitaake plants, but undetectable in *cps2*-KO and *bhlh25*-KO plants (Fig. 3d, e; Supplementary information, Fig. S8c–f). Importantly, *CPS2*-OE plants exhibited a cell death phenotype similar to that observed in *bHLH25*-OE plants (Fig. 3f). When challenged with *M. oryzae*, *CPS2*-OE plants developed smaller disease lesions and harbored less fungal biomass than Kitaake plants, whereas *cps2*-KO plants responded oppositely (Fig. 3g; Supplementary information, Fig. S8g). Moreover, the disease resistance level of *cps2*-KO/*bHLH25*-OE plants was lower than that of *bHLH25*-OE plants (Fig. 3h). Therefore, bHLH25 promotes *CPS2*-mediated phytoalexin biosynthesis to enhance disease resistance.

Interestingly, lignin content remained unchanged in *cps2*-KO and *CPS2*-OE plants (Fig. 3i), in which phytoalexin contents were altered. Similarly, phytoalexin content remained unchanged in *miR397b*-KO and *miR397b*-OE plants, in which lignin contents were altered (Fig. 3j). Thus, bHLH25 promotes disease resistance through two independent defense pathways: *miR397b-*mediated lignin accumulation and *CPS2*-mediated phytoalexin accumulation.

### bHLH25 is directly oxidized at methionine residues by H_2_O_2_

**Fig. 4:**
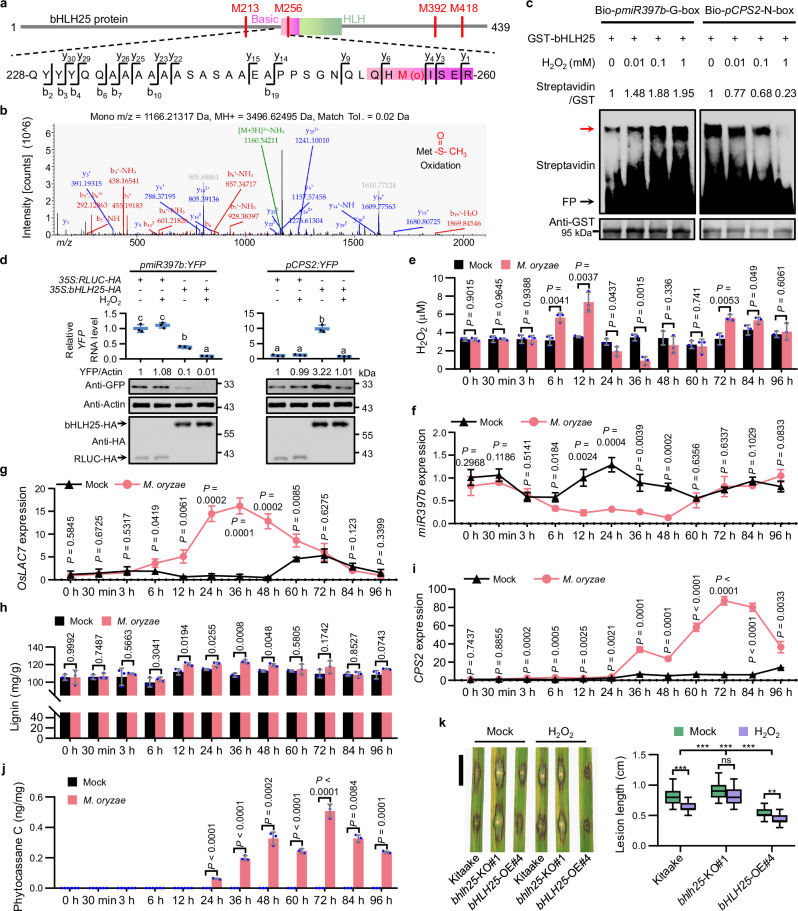
bHLH25 directly senses H_2_O_2_ to regulate *miR397b*- and *CPS2*-mediated defense pathways in rice. **a** Schematic drawing of oxidized methionine residues of bHLH25. M256 of bHLH25 is in the basic domain of bHLH25. Mass-to-charge ratio (*m/z*) is shown. **b** Representative secondary peaks of mass spectrometry spectrum of oxidized M256 of bHLH25. **c** Alteration of bHLH25 DNA-binding specificity by H_2_O_2_ treatment in EMSA. GST-bHLH25 pre-treated with H_2_O_2_ was incubated with probe bio-*pmiR397b*-G-box (left panel) or bio-*pCPS2*-N-box (right panel). The red arrow indicates biotin-labeled probes bound to the GST-bHLH25. **d** H_2_O_2_ enhances the inhibitory effect of bHLH25 on *pmiR397b* (left panels) but weakens the activation effect of bHLH25 on *pCPS2* (right panels) in plants. *YFP* RNA levels (*n* = 3 technical replicates) were determined by RT-qPCR analysis. YFP protein levels, bHLH25-HA and RLUC-HA (negative control) were detected by immunoblot analysis. Signal levels were quantified based on gray-scale values (**c**, **d**). **e**–**j** Endogenous H_2_O_2_ content (**e**, *n* = 3 biological replicates), *miR397b* expression (**f**, *n* = 3 technical replicates), *OsLAC7* expression (**g**, *n* = 3 technical replicates), lignin content (**h**, *n* = 3 biological replicates), *CPS2* expression (**i**, *n* = 3 technical replicates), and phytocassane C content (**j**, *n* = 3 biological replicates) were measured for three-week-old Kitaake leaves 0–96 hpi with or without Zhong10-8-14. **k** Three-week-old Kitaake, *bhlh25*-KO and *bHLH25*-OE plants were pre-treated with or without 1 mM H_2_O_2_ on roots for 72 h, then their leaves were inoculated with Zhong10-8-14. Lesion length (*n* = 30 lesions) at 7 dpi is shown. Scale bar, 1 cm. Data are means ± SD and analyzed by one-way ANOVA with Dunnett’s test (**d**), two-tailed Student’s *t*-test (**e**–**j**) and two-way ANOVA with Tukey’s test at **P < 0.01, ***P < 0.001; ns, not significant (**k**). Experiments were done with three biologically independent replications.

Our previous study showed that post-translational modification in a transcription factor serves as a functional switch to control two independent biological processes.^30^ We therefore performed mass spectrometry analysis on potential post-translational modifications of bHLH25. The results showed that the oxidation level of bHLH25 incubated with extracts of *M. oryzae*-inoculated leaves increased when compared to bHLH25 oxidation level incubated with extracts of mock-treated leaves (Supplementary information, Fig. S9a and Table S7). This oxidation occurred at methionine residues 213, 256, 392, and 418, but not on cysteines, of bHLH25 (Fig. 4a, b).

In addition, we also directly treated bHLH25 protein with H_2_O_2_ in vitro and examined by mass spectrometry analysis the amino acids that became oxidized by H_2_O_2_. The results showed that the oxidation levels at M213/M256/M392/M418 were significantly higher upon direct exposure to H_2_O_2_ than mock (Supplementary information, Fig. S9b and Table S7). These results suggest that bHLH25 is directly oxidized by H_2_O_2_ and upon *M. oryzae* infection.

### Oxidized bHLH25 represses *miR397b* expression and non-oxidized bHLH25 activates *CPS2* expression

DAP-qPCR and EMSA analyses showed that H_2_O_2_ enhanced the binding ability of bHLH25 to the G-box-like-2 motif of *pmiR397b*, but reduced its binding ability to the N-box-like motif of *pCPS2* (Fig. 4c; Supplementary information, Fig. S9c). Transactivation analysis in *N. benthamiana* showed that H_2_O_2_ treatment increased the ability of bHLH25 to repress *pmiR397b* expression, while decreasing the ability of bHLH25 to promote *pCPS2* expression (Fig. 4d). These results indicate that oxidized bHLH25 prefers binding to *pmiR397b* to inhibit *miR397b* expression, while non-oxidized bHLH25 prefers binding to *pCPS2* to promote *CPS2* expression.

During *M. oryzae* infection, H_2_O_2_ was induced at 6–12 hpi (Fig. 4e) and *miR397b* repression and *OsLAC7/28/29* induction occurred at 12–60 hpi in rice plants (Fig. 4f, g; Supplementary information, Fig. S9d, e), which boosted lignin accumulation simultaneously (Fig. 4h). H_2_O_2_ content gradually decreased after 24 hpi due to the activation of lignin biosynthesis, which is supported by our results that H_2_O_2_ levels dropped in *miR397b*-KO and *OsLAC7*-OE plants (Supplementary information, Fig. S9f, g) and by a previous report showing that laccases-mediated lignin polymerization consumes H_2_O_2_.^31^ Consistently, *CPS2* expression increased mostly at 60–96 hpi with *M. oryzae* (Fig. 4i), which boosted phytoalexin accumulation (Fig. 4j).

### bHLH25 and *miR397b* are required for H_2_O_2_-induced disease resistance

Further analyses showed that lignin and phytoalexin induction by *M. oryzae* infection in *bhlh25*-KO plants was obviously delayed and diminished compared to that in Kitaake plants (Supplementary information, Fig. S10a, b). While resistance to *M. oryzae* infection in Kitaake was clearly enhanced by H_2_O_2_ treatment, this enhancement was weakened in *bhlh25*-KO plants, indicating that bHLH25 is required for H_2_O_2_-induced disease resistance; *bHLH25*-OE plants showed a resistance level much higher than Kitaake after H_2_O_2_ treatment (Fig. 4k), supporting the positive role of bHLH25 in H_2_O_2_-induced disease resistance.

*miR397b*-KO plants showed a disease resistance level so high that H_2_O_2_ treatment had no clear effects on *miR397b*-KO plants in disease resistance (Supplementary information, Fig. S10c), indicating that H_2_O_2_ effects on disease resistance are mostly mediated by repression of *miR397b*. Consistently, H_2_O_2_-induced disease resistance was weakened in *miR397b*-OE plants (Supplementary information, Fig. S10c). On the contrary, H_2_O_2_-induced disease resistance was not affected by *CPS2* KO or *CPS2* overexpression in rice plants (Supplementary information, Fig. S10d). These results suggest that the disease resistance induced by H_2_O_2_ treatment requires both *bHLH25* and *miR397b*, but not *CPS2*. Taken together, the oxidation of bHLH25 by H_2_O_2_ is essential for the disease resistance mediated by the *bHLH25*-*miR397b-OsLAC7/28/29* module.

### M256 is essential for bHLH25 function

**Fig. 5:**
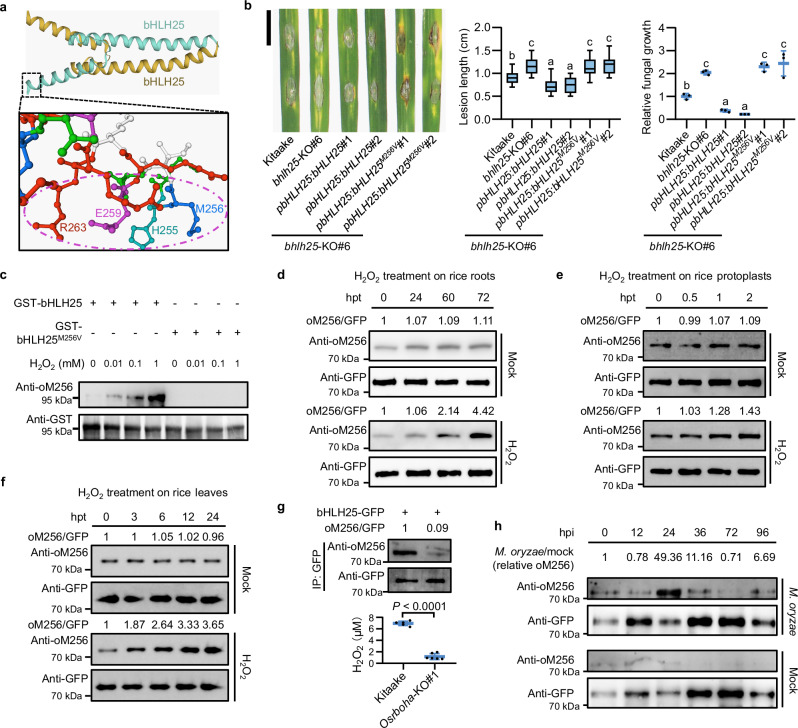
M256 is essential for bHLH25 to sense H_2_O_2_ and promote disease resistance. **a** In silico analysis of the predicted homodimer and the basic domain of bHLH25. The purple circle indicates the basic domain contributing to DNA binding specificity. **b** Representative lesions, lesion length (*n* = 18 lesions) and fungal growth (*n* = 3 technical replicates) of three-week-old Kitaake, *bhlh25-*KO#6, *bHLH25*-complemented and *bHLH25*^*M256V*^-complemented plants 7 dpi with Zhong10-8-14. Scale bar, 1 cm. **c** In vitro oxidation of M256 in bHLH25 in the presence of H_2_O_2_. GST-bHLH25 and GST-bHLH25^M256V^ proteins were treated with 0–1 mM H_2_O_2_ before immunoblotting. Anti-GST antibody indicates GST-bHLH25 and GST-bHLH25^M256V^ levels. **d** In vivo oxidation levels of M256 in bHLH25 in nuclei extracted from three-week-old *bHLH25*-OE#4 plants at different hours post treatment with or without H_2_O_2_. **e**, **f** In vivo oxidation levels of M256 in bHLH25 in nuclei extracted from rice protoplasts (**e**) or leaf clippings (**f**) at different hours post treatment with or without H_2_O_2_. **g** In vivo oxidation levels of M256 in bHLH25 in protoplasts of two-week-old Kitaake and *Osrboha*-KO#1 plants expressing bHLH25-GFP. The endogenous H_2_O_2_ levels in protoplasts were measured (*n* = 6 biological replicates). **h** In vivo oxidation levels of M256 in bHLH25 in three-week-old *bHLH25*-OE#4 plants at different hours post inoculation with or without Zhong10-8-14. Relative oxidation value was calculated by dividing the oxidation value of M256 in bHLH25 treated with *M. oryzae* by that of bHLH25 treated with mock. Anti-GFP detects bHLH25-YFP protein levels immunoprecipitated from rice protein extracts (**f**–**h**). Data are means ± SD and analyzed by one-way ANOVA with LSD test (**b**) and two-tailed Student’s *t*-test (**g**). Experiments were done with three biologically independent replications.

In silico analysis showed that M256 is in proximity to residues H255, E259 and R263 (Fig. 5a) that are key to DNA-binding specificity for bHLH-type transcription factors.^32^ We therefore replaced M256 with comparable hydrophobic residue valine to obtain bHLH25^M256V^ and analyzed the importance of M256. This replacement neither changed the nuclear localization nor the protein stability of bHLH25 in rice (Supplementary information, Fig. S11a, b). Moreover, M256V replacement did not significantly affect the alpha helix structure of bHLH25 basic domain when analyzed by the Self-Optimized Prediction^33^ and Missense3D methods^34^ (Supplementary information, Fig. S11c, d).

EMSA analysis showed that upon H_2_O_2_ treatment, oxidation of bHLH25 enhanced its binding to *pmiR397b*, but weakened its binding to *pCPS2*, whereas bHLH25^M256V^ lost binding to *pmiR397b* and *pCPS2* and was not affected by H_2_O_2_ treatment (Supplementary information, Fig. S11e, f). Unlike *bHLH25* plants, *bHLH25*^*M256V*^ plants failed to repress *miR397b* expression, promoted *CPS2* expression, and enhanced disease resistance to *M. oryzae* (Fig. 5b; Supplementary information, Fig. S11g, i). In addition, *bHLH25*^*M256V*^ plants showed no cell death phenotype (Supplementary information, Fig. S12a, b). Thus, M256 is essential for bHLH25 to regulate *miR397b* and *CPS2* expression and disease resistance.

### The oxidation level of M256 is induced in vivo by H_2_O_2_ treatment and pathogen infection

Immunoblot analysis using a customized antibody (anti-oM256) specifically recognizing oxidized M256 in bHLH25 showed that anti-oM256 antibody detected an increase in oxidized M256 in bHLH25 protein, but no signals in bHLH25^M256V^ protein, after exposure to increased levels of H_2_O_2_ (Fig. 5c). To test the effects of H_2_O_2_ on bHLH25 in vivo, we treated Kitaake roots with H_2_O_2_ and assess bHLH25 RNA levels, protein levels, and M256 oxidation levels. We found no significant changes in *bHLH25* RNA levels (Supplementary information, Fig. S12c). bHLH25 protein levels remained stable when protein extracts from purified nuclei isolated from rice plants treated with H_2_O_2_ were probed in immunoblot analysis; whereas the bHLH25 oxidation level at M256 clearly increased upon H_2_O_2_ treatment in the same in vivo experiment (Fig. 5d). We also performed a similar experiment by directly treating rice protoplasts with H_2_O_2_ and obtained similar results (Fig. 5e). Moreover, direct treatment of rice leaves with H_2_O_2_ significantly increased the oxidation level of bHLH25 and the RNA level of *OsPR1b* in rice leaves (Fig. 5f; Supplementary information, Fig. S12d).

We further tested the H_2_O_2_-sensing ability of bHLH25 in vivo in rice protoplasts by increasing H_2_O_2_ levels with *OsRBOHA*, which encodes an NADPH oxidase responsible for H_2_O_2_ production upon pathogen infection.^35^ Rice protoplasts co-overexpressing bHLH25 and OsRBOHA displayed higher H_2_O_2_ levels and higher M256 oxidization levels than control protoplasts co-overexpressing bHLH25 and HA proteins (Supplementary information, Fig. S12e). Furthermore, protoplasts of *Osrboha*-KO plants showed lower H_2_O_2_ levels and lower M256 oxidization levels than those of wild-type Kitaake plants (Fig. 5g; Supplementary information, Fig. S12f, h). These results suggest that H_2_O_2_ directly targets bHLH25 at M256 in rice nucleus.

In *bHLH25-*OE plants, the M256 oxidation level was highly induced upon *M. oryzae* infection at 12–24 hpi, and started to decrease at ~36 hpi, reaching the lowest level at ~72 hpi. In contrast, mock-treated rice plants did not show obvious oxidation of bHLH25 (Fig. 5h). There is a delay in bHLH25 oxidation compared to H_2_O_2_ level, which may be due to the facts that transport of H_2_O_2_ from other parts of the tissue/cell into the nucleus takes time and that oxidation of bHLH25 also takes time to occur.^36,37^ We also noticed a sharp increase in the total H_2_O_2_ content in rice leaves at 72 hpi with *M. oryzae* (Fig. 4e), while the M256 oxidation level was not increased at this time (Fig. 5h). It is likely that at 72 hpi, H_2_O_2_ mostly accumulates in mitochondria, chloroplasts, and cytoplasm due to cell death induced by phytoalexins^38^ that may not oxidize nuclear bHLH25. Taken together, these results demonstrate that M256 is required for bHLH25 to directly sense H_2_O_2_ upon pathogen infection.

### M256-like methionine residues are highly conserved among bHLH25 orthologues from other plant species

**Fig. 6:**
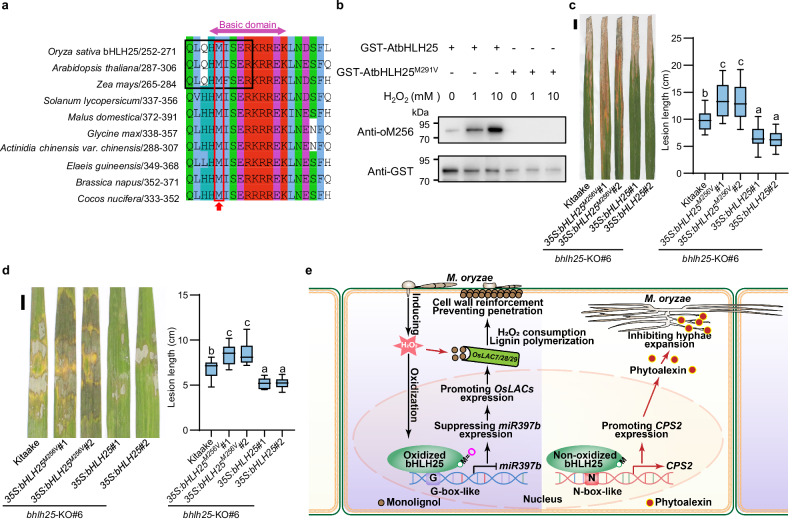
bHLH25 confers resistance to multiple diseases and its M256 is highly conserved among bHLH25 homologs in plants. **a** M256 and neighboring residues in the basic DNA-binding domain of bHLH25 are conserved across different plant species. The red box and arrow highlight the conserved M256-like residues in bHLH25 orthologues, and the black box highlights the target recognized by the oM256 antibody. **b** Immunoblot shows that H_2_O_2_ directly oxidizes the M291 residue of AtbHLH25. GST-AtbHLH25 and GST-AtbHLH25^M291V^ were pre-treated with or without H_2_O_2_ and probed on an immunoblot. Anti-GST indicates a loading control. **c**, **d** Representative lesions and lesion length of tillering-stage Kitaake, and *bHLH25*^*M256V*^- and *bHLH25*-overexpressing plants in *bhlh25*-KO background at 14 dpi with *Xanthomonas oryzae* pv. *oryzae* PXO99A (**c**, *n* = 20 lesions) and at 2 dpi with *Rhizoctonia solani* AG-1-IA (**d**, *n* = 10 lesions). Data are means ± SD and analyzed by one-way ANOVA with LSD test; scale bar is 1 cm (**c**, **d**). **e** Working model for bHLH25 which confers resistance to pathogens by directly sensing H_2_O_2_. H_2_O_2_ induced in rice upon pathogen infection oxidizes bHLH25 at M256 to promote *OsLAC7/28/29* expression, leading to lignin accumulation for cell wall reinforcement to prevent pathogen penetration. Laccases in turn consume H_2_O_2_ in lignin biosynthesis, leading to accumulation of non-oxidized bHLH25. Non-oxidized bHLH25 promotes *CPS2* expression leading to phytoalexin accumulation to inhibit hyphae expansion of pathogens that escape into plant cells. Experiments were done with three biologically independent replications.

To assess the broadness and importance of potential M256-like residues in bHLH25 orthologues, we surveyed all bHLH25 orthologues from 110 plant species that have genome sequences available. Interestingly, M256-like methionine residues are highly conserved in the basic domains of the bHLH25 orthologues from all these 110 plant species (Fig. 6a; Supplementary information, Table S8).

Moreover, immunoblot analysis on *A. thaliana* bHLH25 orthologue (AtbHLH25) showed that its conserved methionine (M291) became oxidized by H_2_O_2_ in wild-type AtbHLH25, but not in AtbHLH25^M291V^ (Fig. 6b), similar to the results of rice bHLH25. Thus, the mechanism by which bHLH25 senses H_2_O_2_ may broadly exist in the plant kingdom.

### bHLH25 confers resistance to multiple diseases caused by fungi or bacteria

Next, we determined the role of bHLH25 in plant biotic and abiotic stress responses. We found that unlike the accumulation of H_2_O_2_ in rice leaves triggered by pathogen infection, which was high and concentrated at and around infection sites, the accumulation of H_2_O_2_ induced by salt and high-temperature stresses was relatively weak and dispersed (Supplementary information, Fig. S13a). Consistently, the oxidation level of bHLH25 was significantly increased in plants upon pathogen infection (Fig. 5h). In contrast, the oxidation level of bHLH25 showed no obvious changes in plants under salt stress or high-temperature stress (Supplementary information, Fig. S13b). More importantly, bHLH25 showed no significant effects on rice resistance to salt stress or high-temperature stress (Supplementary information, Fig. S13c, d). These results suggest that bHLH25 is specifically oxidized by H_2_O_2_ in response to pathogen attack to activate defense responses.

Biotic stresses caused by various pathogens induce H_2_O_2_ production in plants.^3^ We therefore further examined whether bHLH25 confers plant disease resistance to other pathogens, including *R. solani* (a major necrotrophic fungal pathogen to which no effective resistance genes are available), and *Xanthomonas oryzae* pv. *oryzae* (which causes devastating rice bacterial leaf blight).^39,40^ *bHLH25*-OE plants showed smaller lesions, while *bHLH25*^*M256V*^ plants showed larger lesions, than Kitaake plants (Fig. 6c, d). In agreement, *miR397b-*KO plants and *CPS2*-OE plants developed significantly smaller lesions, whereas *miR397b*-OE plants and *cps2*-KO plants developed larger lesions (Supplementary information, Fig. S14). These results indicate that bHLH25 confers resistance to multiple diseases in rice and M256 of bHLH25 is required for this function.

## Discussion

Our study discovers that rice bHLH25 directly senses H_2_O_2_ in the nucleus through M256 to regulate plant disease resistance. In contrast, PRXIIB and QSOX1 may sense cytosolic H_2_O_2_,^14,36^ and HPCA1 senses extracellular H_2_O_2_ through their cysteine residues.^15^ We also uncover that bHLH25 senses H_2_O_2_ to activate two independent defense pathways. Mechanistically, pathogen infection promotes production of H_2_O_2_, which oxidizes bHLH25 at M256 to repress *miR397b* expression, elevating *laccase* expression and lignin biosynthesis to reinforce cell walls to prevent pathogens from penetrating plant cells. Lignin biosynthesis in turn consumes H_2_O_2_ and causes accumulation of non-oxidized bHLH25, allowing bHLH25 to preferentially promote *CPS2* expression to increase phytoalexin biosynthesis, which inhibits growth and expansion of those pathogens that escape into the plant (Fig. 6e). This oxidation/non-oxidation status change of bHLH25 endows plants with an efficient defense system by reinforcing a physical barrier and accumulating antimicrobial phytoalexins. Moreover, this mechanism reveals how plants employ a single protein to coordinate two independent but essential defense pathways.

Excess levels of H_2_O_2_, lignin or phytoalexin cause severe side-effects on plant growth: excess H_2_O_2_ causing oxidation on DNA, RNA, and lipids is toxic to cells^11,41^; elevated lignin levels inhibit plant growth^42–44^; phytoalexin accumulation causes cell death as shown here (Fig. 3a, f) and in previous reports.^45,46^ By using this oxidization/non-oxidization status change of bHLH25, plants homeostatically maintain H_2_O_2_, lignin, and phytoalexin at optimized levels by fine-tuning bHLH25 activity. This precise fine-tuning not only enables plants to effectively defend against pathogen attacks but also prevents the accumulation of these molecules that harm plants.

Our study suggests that high and concentrated accumulation of H_2_O_2_ at and around the pathogen infection sites in plant (Supplementary information, Fig. S13a) leads to clear oxidation of bHLH25 at M256 (Fig. 5h). However, the relatively weak and dispersed H_2_O_2_ accumulation induced by abiotic stresses, like salt and high temperature (Supplementary information, Fig. S13a), might not be able to oxidize bHLH25 or oxidizes bHLH25 at very low level which is not enough for bHLH25 function on lignin biosynthesis (Supplementary information, Fig. S13b). This indicates that plants utilize bHLH25 to sense accumulated high-intensity H_2_O_2_ under pathogen infection and then bHLH25 is oxidized to promote lignin biosynthesis for defending against pathogen attack, while in plants producing relatively dispersed and low-intensity H_2_O_2_ under abiotic stress, bHLH25 cannot be oxidized at an enough level for effectively activating related downstream response. Thus, we speculate that plants may employ bHLH25 to differentiate stimuli from biotic and abiotic stresses through the level of accumulated H_2_O_2_.

Methionine oxidation is an important post-translational modification for protein functions.^47^ Our results showed that M256 oxidation promotes bHLH25 binding to *pmiR397b* but weakens bHLH binding to *pCPS2*. Importantly, the M256V replacement blocks binding of bHLH25 to both *pmiR397b* and *pCPS2* (Supplementary information, Fig. S11e, f). These results are consistent with a previous report showing that the V replacements of ROS-sensing M281/282 in CaMKII abolish the oxidation-dependent kinase activity of CaMKII.^48^ We further performed EMSA to determine the effect of M256 replacement by other amino acids on the DNA binding ability of bHLH25. The results showed that the replacements of M256 in bHLH25 by hydrophobic amino acids (A, P), hydrophilic amino acids (C, T, G, S, N), acidic amino acid (E) and basic amino acid (H) significantly weakened the ability of bHLH25 to bind to *pmiR397b* and *pCPS2* (Supplementary information, Fig. S15). Thus, the replacements of M256 by any other amino acids mainly weaken the DNA binding ability of bHLH25, instead of changing the oxidation state of bHLH25.

Few genes conferring resistance against a wide range of diseases have been identified. As the mechanism used by bHLH25 to sense H_2_O_2_ very likely broadly exists in the plant kingdom (Supplementary information, Table S8) and bHLH25 confers broad-spectrum resistance to diverse diseases caused by fungi or bacteria (Figs. 5b, 6c, d), our study may directly lead to the discovery of a whole class of bHLH25-like transcription factors that confer broad-spectrum disease resistance in other plant species. In *Arabdopsis*, the transcription factor CCA1 HIKING EXPEDITION (CHE) is recently reported to undergo sulfenylation in its conserved cysteine residue in an H_2_O_2_ concentration-dependent manner to promote the expression of salicylic acid-biosynthesis gene to enhance systemic acquired resistance.^49^ Our study, together with this finding, implicate that the Met- and Cys-oxidation mechanism may broadly be employed by eukaryotic transcription factors to sense H_2_O_2_ to change their roles. Thus, our findings may have great impacts on the research field of H_2_O_2_ signaling in both plants and animals.

## Materials and methods

### Plant materials and growth conditions

The full-length cDNA sequences of *OsLAC7* (*Os01g0850550*), *OsLAC28* (*Os12g0257600*), *OsLAC29* (*Os12g0258700*), *CPS2* (*Os02g0571100*), *bHLH25* (*Os01g0196300*) and *OsRBOHA* (*Os01g0734200*) were cloned from mRNAs of Nipponbare rice. For generating CRISPR/Cas9 vectors to knock out single genes, 20 bp target sequences were selected by online tool (http://crispr.hzau.edu.cn/CRISPR2/), and were cloned into the BGK032-Cas9 vector. For generation of double KO of *OsLAC7/29* and triple KO of *OsLAC7/28/29*, we cloned the target sequences into the pTCRISPR-Cas9 vector. For generating the construct for *pbHLH25:YFP*, the 1500 bp native promoter of *bHLH25* was amplified from Nipponbare DNA to replace the *35S* promoter in pCAMBIA1300–*35S:YFP*. To study M256 for bHLH25 function, we generated mutant bHLH25^M256V^ in which M256 of bHLH25 was replaced by a valine, which is a hydrophobic amino acid commonly used for amino acid substitution to minimize structural changes as previously reported.^48^ For generating vectors overexpressing genes other than *bHLH25*, including *miR397b*, *CPS2* and *OsLAC7/28/29*, we cloned the precursor of *miR397b (pre-miR397b)* DNA with extended 300 bp toward both 3’ and 5’, and the cDNA sequences of *CPS2*, *OsLAC7*, *OsLAC28* and *OsLAC29* into the *35S* promoter-driven overexpression vector individually.

For generating transgenic plants, vectors were transferred into *Agrobacterium tumefaciens* EHA105 as previously reported.^50^ EHA105 strains containing vectors were used to transform Kitaake background (*Oryza sativa japonica*). *bHLH25* KO plants were transformed with constructs carrying *bHLH25* or *bHLH25*^*M256V*^, driven by a native *bHLH25* promoter or driven by *35S* promoter to determine the importance of M256 for bHLH25. For genes other than *bHLH25*, including *miR397b*, *CPS2* and *OsLAC7/28/29*, we made their single, double, or triple KO plants to verify their roles in biosynthesis of lignin or phytoalexin and disease resistance, and introduced the corresponding overexpression constructs of these genes into wild-type Kitaake plants to make their overexpression plants. The *Oslac7/28/29*-KO/*miR397b*-KO plants were created by crossing *Oslac7/28/29*-KO#1 and *miR397b*-KO#1. The *miR397b*-OE/*bHLH25*-OE plants were created by crossing *miR397b*-OE#1 and *bHLH25*-OE#4. The *cps2*-KO/*bHLH25*-OE plants were created by crossing *cps2*-KO#1 and *bHLH25*-OE#4.

Rice plants were grown in the experimental field of the Sichuan Agriculture University (Chengdu, Sichuan, China). We used PCR to amplify the Cas9-edited target sequences for KO plants and sequenced the PCR products to ensure that the plants we used for experiments were progeny of sequenced stable homozygous plants. For H_2_O_2_ treatment and *M. oryzae* spray inoculation assays, plants were grown with Yoshida’s nutrient solution in an incubator or grown in a greenhouse under a short-day photoperiod (11 h light/13 h dark) with light strength of approximately 20,000 lux at 28 °C/22 °C (day/night) cycles.^51^

### H_2_O_2_ treatment and H_2_O_2_ content measurement

For treating roots of rice plants with H_2_O_2_, we transferred three-week-old seedlings of Kitaake from the basal nutrition solution to nutrient solution containing 1 mM H_2_O_2_ or water mock for 0–72 h, then used treated leaves from the same part of plants for further detection as previously described.^52^ For treating rice leaves with H_2_O_2_, we clipped rice leaves to 1 cm^2^ and treated them with 1 mM H_2_O_2_ or mock solution. For treating rice protoplasts with H_2_O_2_, we added water or H_2_O_2_ to protoplasts of two-week-old rice seedlings to 0 or 1 mM H_2_O_2_ and the treatment lasted for 0–2 h. For treating protein with H_2_O_2_ in vitro, we incubated 10 μg purified GST-tagged protein with different concentrations of H_2_O_2_ in 300 μL solution at 28 °C for 15 min.^53^

For measuring the total H_2_O_2_ contents of rice plants, we finely ground ~20 mg of each rice leaf sample or used 0.5 mL rice protoplasts for further measurement using a hydrogen peroxide assay kit (Beyotime, #S0038) following the manufacturer’s instructions and previous studies.^52,54^ This assay is based on the formation of ferric ion–xylenol orange complexes from xylenol orange and ferrous ion upon oxidation by H_2_O_2_. As H_2_O_2_ accumulation, expression levels of *PR* genes and *OsLAC7/28/29* genes were most profound at 60 and 72 hpt with H_2_O_2_, we therefore focused on these two time points for examining rice gene expression in rice leaves and disease resistance after treatment with H_2_O_2_. These experiments were done with three biologically independent replications.

### Plant disease resistance assay

For inoculation with *M. oryzae*, different strains of Zhong10-8-14 and ZB25 were used to verify disease resistance of rice plants. For punch inoculation assays, rice leaf strips of three-week-old seedlings were punched, inoculated with 5 μL spore suspension (1 × 10^5^ spore/mL) of *M. oryzae*, and incubated at 28 °C as previously described.^55^ Lesion length was measured 7 days after inoculation. For relative fungal growth measurement, DNA was extracted from infected leaves to examine the relative DNA level of the *Pot2* gene (*AF314096*) of *M. oryzae*, relative to rice *ubiquitin* (*Os03g0234200*), by qPCR analysis.^56^ For spray inoculation assays, leaves of three-week-old seedlings were sprayed with spore suspension of *M. oryzae*.^57^ Then the leaves from the same part of the plants inoculated with *M. oryzae* were taken for further measurement of H_2_O_2_ content, lignin, phytocassane C, and gene expression at time points from 0–96 h.^55,58^ For determination of plant disease resistance by lesion measurement in spray inoculation, the number of lesions were counted for each infected leaf for 7 days after *M. oryzae* inoculation.

For inoculation with *R. solani*, fully expanded leaves of tillering-stage rice plants were inoculated with mycelial clumps of the AG-1-IA strain following a previously reported method.^59^ For inoculation with *Xanthomonas oryzae* pv. *oryzae*, fully expanded leaves of tillering-stage rice plants were cut at about 1 cm from the leaf tip using a pair of scissors pre-dipped in the bacterial suspension of strain PXO99A (OD600 = 0.6) following a previously reported method.^19^ The spontaneous cell death phenotype of *bHLH25*-OE plants is completely different in appearance from the disease lesions in rice leaves caused by pathogen infection, therefore the cell death phenotype does not affect the determination of disease resistance in plants. Three biologically independent replications were performed for all plant disease assays.

### RNA extraction, RT-qPCR, semi-qPCR and RNA-seq analysis

For RNA extraction to perform RT-qPCR and RT-semi-quantitative PCR (RT-semi-qPCR), samples were frozen and finely ground in liquid nitrogen and total RNA was extracted using TRIzol (Invitrogen, #15596018). Gene transcript levels were determined by RT-qPCR (QIAGEN, #208054) or RT-semi-qPCR (Vazyme, #P505-d1). The first strand cDNA was synthesized using a reverse transcription kit (Takara, #RR047B). At least three biologically independent replications were performed. To specifically detect the expression of *miR397b*, we designed primers to examine *pre-miR397b* levels. Amplification cycles were normalized against rice *ubiquitin*, or *NbEF1α* in *N. benthamiana*,^60^ by calculating differences between the threshold cycle (C_T_) of the target gene and the C_T_ of *UBQ5* or *NbEF1α*. Relevant primer sequences are given in Supplementary information, Table S9.

For RNA-seq analysis, total RNA extracted from leaves of three-week-old rice plants at 60 and 72 hpt with or without H_2_O_2_ were used to construct Illumina sequencing libraries (NEB, #102715-922). Illumina sequencing libraries were sequenced using the Illumina system Hiseq4000 with 250–300 bp read lengths (Novogene, Bejing, China). The paired-end clean reads of RNA-seq were aligned to the *Oryza sativa* IRGSP 1.0 genome DNA using the HISAT2 software. We performed quantitative analysis of gene expression level for each sample, then combined them to obtain expression matrix for all samples. Analysis of differentially expressed genes (DEGs) was conducted using the edgeR software,^61^ and statistical analysis was performed by Hypergeometric test with Bonferroni correction. The enriched functions of DEGs in RNA-seq data were annotated with the gene ontology function in the clusterProfile and agriGOv2^62^ software.

### Promoter analysis

Conserved *cis*-elements in the promoter regions of *OsLAC7/28/29* were identified in the 1000 bp flanking sequences upstream of the transcription start sites of *OsLAC7/28/29*. The promoter sequences were analyzed by the PlantCARE^63^ online tool (http://bioinformatics.psb.ugent.be/webtools/plantcare/html/) to predict *cis*-elements.

### MBS analysis

To decipher miRNAs regulating the expression of *OsLAC7/28/29*, we used the psRNATarget online tool (https://www.zhaolab.org/psRNATarget/) to predict potential MBSs^64^ in mRNAs of *OsLAC7/28/29*. MBSs were selected with a miRNA binding expectation value less than 0.5.

### Transactivation assay in *N. benthamiana*

Transactivation assays were performed as previously reported.^65^ The *Agrobacterium* strain GV3101 was used for transformation of *N. benthamiana*. For generating reporter plasmids for regulation by *miR397b*, we fused the *miR397b* MBS or mutated MBS (mMBS) of *OsLAC7/28/29* with YFP in the pCAMBIA1300-*35S:YFP* vector. *35S:miR397b* was used as the effector. For generating reporters for regulation by bHLH25, we amplified the 1000 bp regions of *pmiR397b* and *pCPS2*, then inserted them into the pCAMBIA1300-*35S:YFP* vector to replace *35S* promoter individually. *35S:bHLH25-HA* acted as an effector, and *35S:Renilla luciferase* (*RLUC*)-HA acted as a negative control. For transformation, GV3101 strains containing constructs in MMA buffer (10 mM MES, 10 mM MgCl_2_, 100 µM AS) at OD600 = 1 were infiltrated into leaves of *N. benthamiana* and incubated for 36 h, followed by injection of different concentrations of H_2_O_2_ and incubation for another 12 h. 0.5 g leaves were harvested, followed by extraction of RNAs for RT-qPCR analysis or proteins for immunoblot analysis. Each set of data were generated with three biologically independent replications and derived from the total protein extracts obtained from the same *N. benthamiana* leaf.

To determine the transcriptional regulation of *OsLAC7/28/29* by TBPs and CBFs, we only successfully obtained the CDS of 18 of 25 TBPs and CBFs, and then cloned them into the pGreenII-62SK vector driven by *35S* promoter to generate effector constructs. The 1000 bp promoter regions of *OsLAC7*, *OsLAC28* and *OsLAC29* were separately cloned into the pGreenII-0800 to drive firefly luciferase (LUC) expression. *Renilla luciferase* (*RLUC*) driven by *35S* promoter was used as a reference. The empty pGreenII-62SK vector was used as a negative control. Rice protoplasts were transfected with different combinations of plasmids and incubated overnight, then collected and lysed for determining relative RNA levels of *LUC* and *RLUC* by RT-qPCR analysis. Relevant primer sequences are given in Supplementary information, Table S9.

### Yeast one-hybrid assay

For generating the bait vector for yeast one-hybrid library screening, the 1000 bp *pmiR397b* was fused to the *HIS3* reporter in pHIS2 vector. The prey cDNA library, constructed from rice seedlings of Digu inoculated with *M. oryzae*, was prepared in the pGADT7-*Rec2* vector (Clontech) and transformed into yeast strain AH109 (Clontech) as described previously.^55^ Yeast diploids were selected by incubating at 30 °C for 4 days on minimal medium SD lacking Trp, Leu (-T-L) or lacking Trp, Leu, His (-T-L-H) while supplemented with 30 mM 3-amino-1,2,4-triazole (3-AT, Sigma-Aldrich, #A8056). Positive clones were sequenced and confirmed. For confirming bHLH25 DNA binding activity, the CDS of *bHLH25* was amplified and cloned into the pGADT7-*Rec2* vector fusing to the activation domain. Constructs were co-transformed into AH109. The empty vector pGADT7-*Rec2* was co-transformed as a negative control. DNA–protein interactions were determined by the growth of transformants on the nutrient-deficient medium with 30 mM 3-AT. Relevant primer sequences are given in Supplementary information, Table S9. Three biologically independent replications were performed.

### Rice protoplast transient expression analysis

Protoplasts of two-week-old etiolated Kitaake seedlings were isolated and transformed with constructs following the method as described previously.^66^ For subcellular localization analysis, we generated constructs expressing bHLH25-GFP from vector pRTVcGFP (*Ubi:GFP*).^67^ Constructs for *Ubi:GFP* or *Ubi:bHLH25-GFP* were co-transformed with nuclear marker *pSAT6:RFP-VirD2NLS* respectively into protoplasts. Fluorescence was examined under a confocal microscope at 16 h post transformation. For testing whether bHLH25 senses H_2_O_2_ produced by NADPH oxidase in vivo, we generated a construct expressing immunity-related NADPH oxidase gene *OsRBOHA* by cloning it into vector pRTVcHA (*Ubi:4*×*HA*). About 1 μg plasmid was used to transform 100 μL protoplasts (2 × 10^6^ protoplast/mL) by incubating at 28 °C for 16 h. Relevant primer sequences are given in Supplementary information, Table S9. Three biologically independent replications were performed.

### Protein purification in vitro and extraction in vivo

For generating vectors expressing GST-tagged wild-type and mutant bHLH25 proteins in vitro, CDSs of *bHLH25* and and its mutant forms were individually cloned into the pGEX-6p-1-small ubiquitin-like modifier (SUMO) vector. To investigate function of the *A. thaliana* bHLH25 orthologous protein, CDSs of *AtbHLH25 (AT5G56960)* and *AtbHLH25*^*M291V*^ were individually cloned into the pGEX-6p-1 vector. The constructs were transformed into the *E. coli* strain Transetta (DE3) (TransGen Biotech, #CD801-02). Relevant primer sequences are given in Supplementary information, Table S9. Transetta strains were grown in LB medium containing 100 mg/mL ampicillin at 37 °C to OD600 = 0.6. The expression of the fusion proteins was induced by adding IPTG (Sigma, #I6758) to 1 mM and incubating at 28 °C for 14 h. The fusion proteins were purified with glutathione sepharose 4B beads (GE, #17075601).

For extracting protein in vivo, total protein was extracted with native protein extraction buffer (50 mM Tris-MES at pH 8.0, 0.5 M sucrose, 1 mM MgCl_2_, 10 mM EDTA, 5 mM DTT and protease inhibitor cocktail (TransGen Biotech, #DI111-01)). For bHLH25-YFP immunoprecipitation (IP), we used the same amount of GFP-trap agarose beads (Chromotek, #gta-100) to enrich bHLH25-YFP protein in leaves of the same weight at each time point. The result of immunoblot analysis showed that the level of bHLH25-YFP protein extracted from rice was indeed different at different time points, which may be due to the different protein turnover rates at different time points that regulate bHLH25 protein level. Plasma membrane protein OsRBOHA-4×HA and nuclear protein bHLH25 were extracted by differential centrifugation according to the manufacturer’s instruction of Plasma Membrane Protein and Nuclei Isolation Kit (Invent, #PF-045).

### DAP-qPCR analysis

DAP assay was performed with three biologically independent replications as previously described.^55^ In brief, total DNA was extracted from three-week-old Kitaake seedlings and sonicated into 100–500 bp fragments by using ultrasonic crusher. DNA fragments, GST-tagged protein, and glutathione-agarose were co-incubated in incubation buffer (50 mM Tris-HCl, pH 7.0, 1 mM EDTA, 100 mM KCl, 5% Glycerol, 0.1% Triton X-100, 1 mM DTT) at 4 °C for 2 h. After co-incubation, glutathione-agarose beads were washed three times with incubation buffer. Then 4 mL 5 M NaCl was added into the sample for each 100 mL volume and incubated at 65 °C for 4 h to break down cross-linked GST-tagged protein and DNA fragments. Then extracted DNA was applied for qPCR using respective primer pairs. The *ubiquitin* promoter was used as the internal control. Relevant primer sequences are given in Supplementary information, Table S9.

### ChIP-qPCR analysis

Four-week-old *35S:bHLH25*#1 (in *bhlh25*-KO#6 background) plants were used for ChIP analysis according to methods described previously.^30^ Protein A magnetic beads (Sangon Biotech, #D110560) with no antibody (No Abs) were used as a negative control. Relevant primer sequences are given in Supplementary information, Table S9. Three biologically independent replications were performed.

### EMSA

The detailed procedure of EMSA follows instructions of Light Shift Chemiluminescent EMSA Kit (Thermo Scientific, #20148). Biotin was labeled at the 5’ end of oligonucleotides containing *cis*-elements. For pre-treating bHLH25 proteins with H_2_O_2_, we used glutathione sepharose 4B beads to pull out bHLH25 protein pre-treated with H_2_O_2_. We then incubated the pulled-out bHLH25 protein with probes in reaction solution free of H_2_O_2_ to perform EMSA. The components of 10 μL binding reaction include 10 mM Tris-HCl (pH 7.5), 50 mM KCl, 50 ng/mL Poly (dI·dC), 1 mM DTT, about 0.5 μg purified GST-tagged proteins, 0.5 μM *pmiR397b*-G-box or 0.1 μM *pCPS2*-N-box biotin-labeled DNA. Each dataset consisted of three biologically independent replications.

### Lignin measurement

Lignin measurement was performed as reported previously with some adjustments with three independent biological replications.^68^ Briefly, fresh samples (0.5 g) were ground in liquid nitrogen and re-suspended in 7 mL ethanol. The sample was pelleted by centrifugation (1400× *g*, 5 min) and the pellet was extracted with the following solvents for 15 min each: two times with 7 mL ethanol, three times with 7 mL distilled water, two times with 7 mL ethanol/n-hexane (1:2 [v/v]), and once with 5 mL ethanol. The pellet was dried at 60 °C for 72 h. Subsequently, 20 mg of dry pellet was added to 0.5 mL of 25% acetyl bromide (v/v in glacial acetic acid) and incubated at 70 °C for 30 min. After quick cooling on ice, the sample was dissolved in 6 mL solution containing 0.9 mL 2 M NaOH, 0.1 mL 7.5 M hydroxylamine-HCl and 5 mL of glacial acetic acid. The absorbance of the supernatant was measured at 280 nm after centrifugation (1400× *g*, 5 min). Lignin content was calculated using the Beer-Lambert law (*A* = *Kbc*).

### Histochemical staining assay

For histochemical staining of lignin, we used phloroglucinol-HCl staining as described before.^69^ In brief, leaves of four-week-old rice plants were sectioned with a razor blade. Sections were stained with 1% phloroglucinol (w/v) in 12% HCl for 5 min and immediately observed under a stereo microscope (SMZ1000, Nikon, Japan). The thickness of sclerenchyma cells was quantitated as described previously.^18^ These experiments were done with three biologically independent replications.

### Phytoalexin measurement

Phytoalexin content was measured with three biologically independent replications as described previously.^29^ In brief, rice leaf samples (10 mg fresh weight) were soaked in 1 mL of extraction solvent (MeOH/H_2_O, 80:20 [v/v]) in glass tubes and incubated at room temperature overnight. The extract was centrifuged (4 °C, 15 min, 16,000× *g*). The supernatants were then collected and dried up. Finally, dried samples were subjected to phytoalexin measurement by LC-ESI-MS/MS as described before.^70^

### TEM analysis

Leaf segments from four-week-old rice plants were transferred into plastic tubes with fresh TEM fixative (Servicebio, #G1102) for fixation under vacuum extraction until the samples sink to the bottom. Samples were treated according to a previous study.^71^ Samples were observed in TEM Hitachi HT7800. Sclerenchyma cell wall thickness was calculated with ImageJ. These experiments were done with three biologically independent replications.

### Mass spectrometry analysis of bHLH25 oxidation

For identifying post-translational modifications on bHLH25 protein after *M. oryzae* infection, we used extraction buffer (50 mM Tris-MES at pH 8.0, 0.5 M sucrose, 1 mM MgCl_2_, 10 mM EDTA, 5 mM DTT and protease inhibitor cocktail) to obtain cell extracts from Kitaake rice leaves treated with *M. oryzae* or mock control for 24 h. Then we incubated purified GST-bHLH25 protein with cell extracts at 4 °C for 4 h. Finally, the GST-bHLH25 protein was purified using glutathione sepharose 4B beads (GE, #17075601) and used for mass spectrometry analysis.

The stained protein band was cut, and the volume of the cut gel was 0.5–1 mm^3^. The cut gel was de-stained with 50% acetonitrile (ACN) in 50 mM triethylammonium bicarbonate (TEAB) and dehydrated upon washing with 100% ACN till the gel turned white. Proteins in gel were treated with 1 mL of 10 mM DTT for 40 min at 56 °C and subsequently alkylated with 1 mL of 50 mM IAM for 30 min in the dark. The gel was washed with de-staining buffer and treated with ACN as above. Protein digestion: add 10–20 µL 10 ng/µL pancreatic enzyme, rest on ice for 30 min, and wait for the enzyme solution to be completely absorbed by the colloidal particles, add 100 mM TEAB buffer to the total volume of 100 µL, and allow enzyme digestion at 37 °C overnight. The supernatant was collected by centrifugation at low speed, 100 µL 0.1% formic acid was added into the glue, and the supernatant was collected by centrifugation at low speed after oscillating at room temperature for 5 min. Sample cleanup: centrifuge at room temperature 12,000× *g* for 5 min, take the supernatant slowly through the C18 desalting column, and then wash it with 1 mL cleaning solution (0.1% formic acid, 4% acetonitrile) for 3 consecutive times, then add 0.4 mL eluent (0.1% formic acid, 75% acetonitrile) for 2 consecutive times, and then freeze dry the elution sample after combination.

Mobile phase A (100% water, 0.1% formic acid) and B solution (80% acetonitrile, 0.1% formic acid) were prepared. The lyophilized powder was dissolved in 10 μL of solution A, and centrifuged at 15,000 rpm for 20 min at 4 °C. 1 μg of sample was injected into a C18 Nano-Trap column. Peptides were separated in an analytical column, using a linear gradient elution. The separated peptides were analyzed using a Q Exactive HF-X mass spectrometer (Thermo Fisher), with ion source of Nanospray Flex (ESI), spray voltage of 2.3 kV, and ion transport capillary temperature of 320 °C. Full scan range from *m/z* 350 to 1500 with resolution of 60,000 (at *m/z* 200), an automatic gain control (AGC) target value was 3 × 10^6^ and a maximum ion injection time was 20 ms. The top 40 precursors of the highest abundance in the full scan were selected and fragmented by higher energy collisional dissociation and analyzed in MS/MS, where resolution was 15,000 (at *m/z* 200), the AGC target value was 1 × 10^5^, the maximum ion injection time was 45 ms, a normalized collision energy was set as 27%, an intensity threshold was 2.2 × 10^4^, and the dynamic exclusion parameter was 20 s. The resulting spectra from each fraction were searched against Oryza_sativa_subsp_japonica_uniprot_2021_3_9.fasta database separately by Proteome Discoverer 2.2. The identified protein contains at least 1 unique peptide with FDR no more than 1.0%. Proteins containing similar peptides that could not be distinguished by MS/MS analysis were identified as a same protein group. Oxidation of residues was specified in Proteome Discoverer 2.2 as variable modifications. These experiments were done with three biologically independent replications.

### Immunoblot analysis

Proteins were electrophoresed on a 10% SDS-PAGE gel before being transferred to a PVDF membrane (Merck Millipore, Billerica, MA, USA) using a Trans-Blot® SD Semi-Dry Transfer Cell (Bio-Rad, Hercules, CA, USA) according to standard procedures. For detecting YFP and GFP proteins, we used anti-GFP as previously reported.^72^ For detecting biotin, we used streptavidin-HRP (Invitrogen, #434323). The monoclonal antibody against oxidized M256 of bHLH25 (anti-oM256) was generated by ABclonal Technology (Wuhan, China). We used an oxidized bHLH25 short peptide (QLQH-(oxidized M)-ISER) to immunize mice and screen for positive hybridoma clones, and a non-oxidized bHLH25 short peptide (QLQH-(M)-ISER) to exclude any hybridoma clones generating antibodies that reacted with the non-oxidized peptide, in enzyme-linked immunosorbent assay (ELISA) and immunoblot analysis. Relative levels of band gray-scale values were measured by Image Lab 3.0 software (Bio-Rad). Relative oxidation value was calculated by dividing the oxidation value of bHLH25 treated with *M. oryzae* by that of bHLH25 treated with mock. The antibodies used in this study: anti-GFP (Invitrogen, #PA1-980A, 1:10,000), anti-HA (Invitrogen, #26183, 1:5000), anti-Actin (Sangon Biotech, #D110007, 1:2000), anti-GST (Invitrogen, #A-5800, 1:5000), anti-Rabbit IgG HRP (Invitrogen, #31460, 1:10,000), anti-Mouse IgG HRP (Invitrogen, #62-6520, 1:10,000), and anti-oM256 (1:1000). All experiments were conducted with three biologically independent replications.

### In silico analysis

For predicting the structure of bHLH25 protein, we used the SWISS-MODEL (https://swissmodel.expasy.org/)^73^ or Alphafold (https://www.alphafold.ebi.ac.uk/).^74,75^ For the prediction by SWISS-MODEL, bHLH transcription factor MYC2 was selected as the most suitable template for bHLH25 to calculate and simulate the structure of bHLH25. For evaluating the structural effect of bHLH25^M256V^ on alpha-helix structure in the basic domain of bHLH25, we performed the Missense3D analysis^34^ based on the structures of bHLH25 and bHLH25^M256V^ proteins predicted by Alphafold2. For evaluating the structural effect of bHLH25^M256V^ on the protein secondary structure of bHLH25, we conducted self-optimized prediction method (SOPMA)^33^ on Network Protein Sequence Analysis^76^ to analyze the alpha-helix structure in the basic domain of bHLH25.

### Protein stability determination

Protein stability was determined by immunoblot analysis according to a previous study.^77^ Plasmids harboring bHLH25-GFP or bHLH25^M256V^-GFP were used to transform rice protoplasts. After incubation for 12 h, protoplasts were treated with 100 mM protein synthesis inhibitor cycloheximide for different time periods (time points). Three biologically independent replications were performed.

### Orthologue analysis

The protein accessions and detailed information of bHLH25 orthologues in the plant kingdom were obtained from the Basic Local Alignment Search Tool of the National Center for Biotechnology Information (https://blast.ncbi.nlm.nih.gov/Blast.cgi). The alignment was visualized using JalView and colored by ClustalX. All protein accessions used for analysis and other M256 orthologues of bHLH25 in the conserved basic domains were shown in Supplementary information, Table S8.

### High-temperature treatment

High-temperature treatment was performed with three biologically independent replications according to a previous report.^78^ In brief, three-week-old seedlings were treated at 42 °C in growth chambers for three days, then they were returned to normal conditions (26 °C) for 5-day recovery.

### Salt stress treatment

Salt stress treatment was performed with three biologically independent replications according to a previous report.^79^ Salt stress was initiated 3 h after the start of the light period by incubating 3-week-old seedlings in nutrition solution with 150 mM NaCl for three days, then they were returned to normal nutrition solution for 5-day recovery.

### 3,3-diaminobenzidine (DAB) staining

DAB staining was conducted with three biologically independent replications as described previously.^77^ Rice leaves were submerged in staining buffer (10 mM MES, pH 6.5 and 1 mg/mL DAB) for 18 h in darkness at room temperature and transferred to 90% (v/v) ethanol at 65 °C until all the chlorophyll had been removed. Then the cleared leaves were photographed.

### Quantification and statistical analysis

For statistical analysis, the two-tailed Student’s *t*-test was performed by using the Office Excel software; the one-way ANOVA with LSD and Dunnett’s multiple comparison test at *P* < 0.05 was performed by using the IBM SPSS software (v.21); the two-way ANOVA with Tukey’s multiple comparison test at *P* < 0.05 was performed by using R (v.4.4.0). Data were presented as means ± SD as indicated. *P* values over 0.05 were considered not significant. Sample sizes, statistical tests used and *P* values are stated in the figures and figure legends.

## Supplementary information

Fig. S1

Fig. S2

Fig. S3

Fig. S4

Fig. S5

Fig. S6

Fig. S7

Fig. S8

Fig. S9

Fig. S10

Fig. S11

Fig. S12

Fig. S13

Fig. S14

Fig. S15

Supplementary information, Table S1

Supplementary information, Table S2

Supplementary information, Table S3

Supplementary information, Table S4

Supplementary information, Table S5

Supplementary information, Table S6

Supplementary information, Table S7

Supplementary information, Table S8

Supplementary information, Table S9

## Data Availability

All materials are available upon request subject to a material transfer agreement. All data are available in the main text or the supplementary information. The RNA-seq data for analyzing H_2_O_2_-triggered gene expression have been deposited into the NCBI Short Read Archive under accession PRJNA786258.
