# Supplementary material for: Rice transcription factor bHLH25 confers resistance to multiple diseases by sensing H2O2
Source: Cell Res. 2025 Jan 14;35(3):205–19. doi: 10.1038/s41422-024-01058-4 (PMC11909244; doi:10.1038/s41422-024-01058-4)
Supplement: Supplementary file 1 — Fig. S1 [file 41422_2024_1058_MOESM1_ESM.pdf]

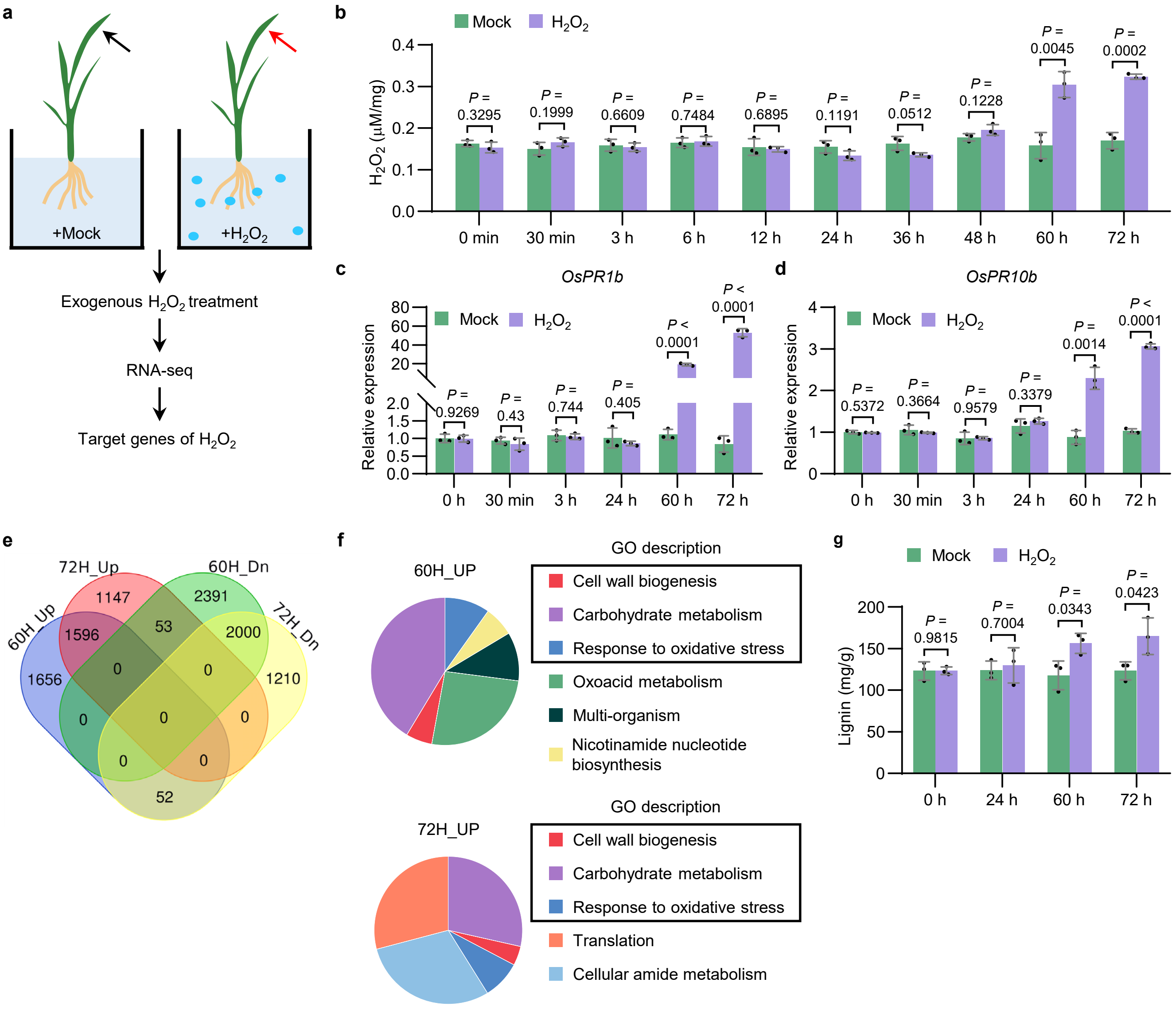

Supplementary information, Fig. S1 H_2O_2 triggers immunity and cell wall-related biological process. a Workflow for identifying target genes of H_2O_2. **b** H_2O_2 content in leaves of three-week-old Kitaake treated with or without 1 mM H_2O_2 on roots for 0-72 h (mean ± s.d., *n* = 3 technical replicates). **c**, **d** RNA levels of *OsPR1b* (**c**) and *OsPR10b* (**d**) in three-week-old Kitaake 0-72 hpt with 1 mM H_2O_2 (mean ± s.d., *n* = 3 technical replicates). Mock treatment indicates without H_2O_2. **e** Pairwise comparisons of up-regulated and down-regulated genes in three-week-old Kitaake plants treated with H_2O_2 for 60 h and 72 h, on the threshold of fold change >1.5 and $P < 0.05$. **f** Gene ontology analysis of significantly upregulated genes in three-week-old Kitaake at 60 and 72 hpt with H_2O_2. The black box indicates biological processes induced in rice at both 60 and 72 hpt with H_2O_2. **g** Lignin contents of three-week-old Kitaake plants at 0-72 hpt with 1 mM H_2O_2 (mean ± s.d., *n* = 3 biological replicates). Data were analyzed by two-tailed Student's *t*-test (**b-d**, **g**). Experiments were done with three biologically independent replications.
