# Supplementary material for: Rice transcription factor bHLH25 confers resistance to multiple diseases by sensing H2O2
Source: Cell Res. 2025 Jan 14;35(3):205–19. doi: 10.1038/s41422-024-01058-4 (PMC11909244; doi:10.1038/s41422-024-01058-4)
Supplement: Supplementary file 2 — Fig. S2 [file 41422_2024_1058_MOESM2_ESM.pdf]

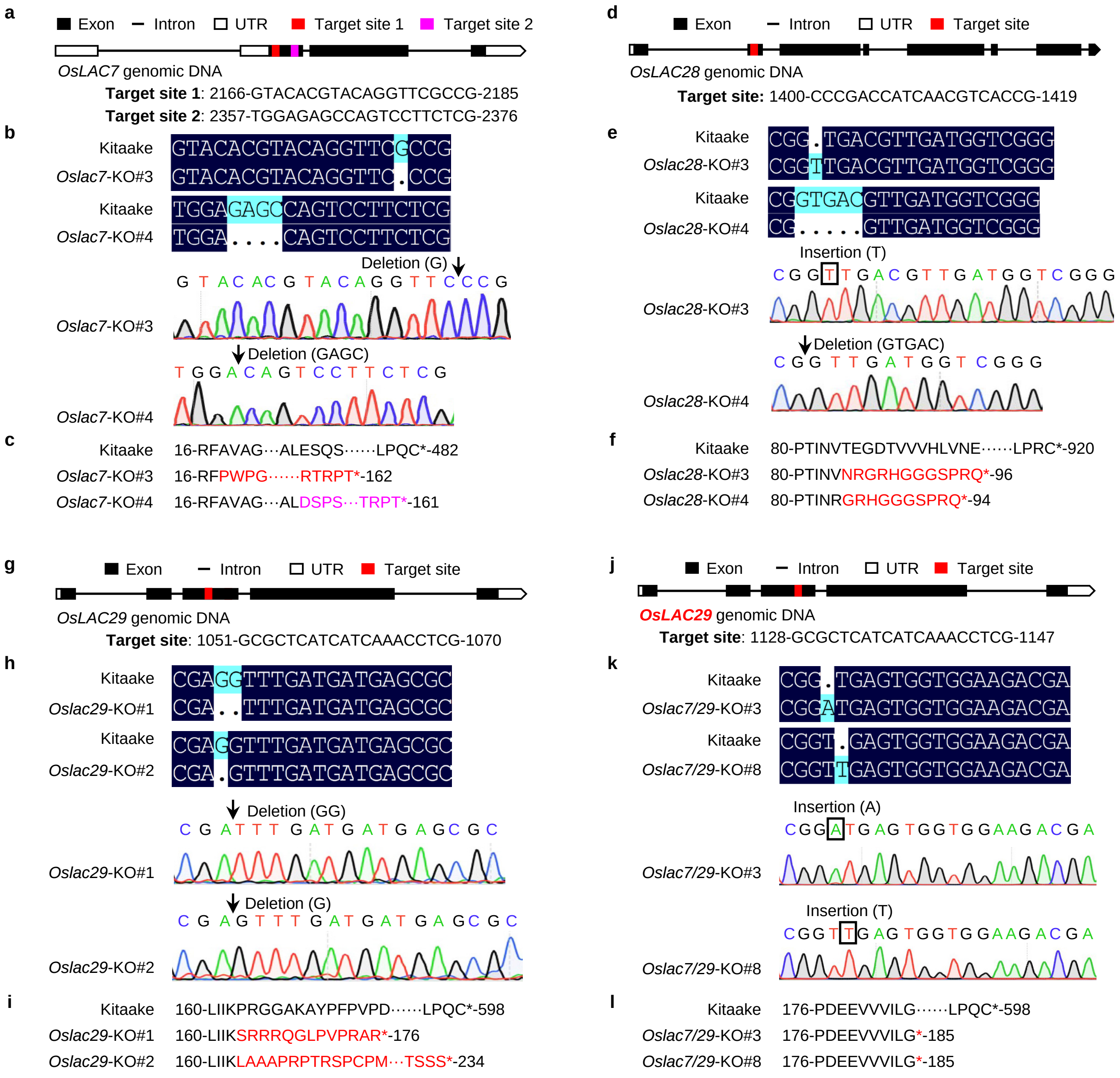

Supplementary information, Fig. S2 Generation of single knockout (KO) for each of *OsLAC7/28/29* and double KO plants of *OsLAC7/29*. a Schematic drawing of two target sites designed for knocking out *OsLAC7*. **b** Verification of two independent *Oslac7*-KO lines (*Oslac7*-KO#3 and *Oslac7*-KO#4) by PCR-based sequencing. **c** The alignment of OsLAC7 amino acid sequences encoded in Kitaake, *Oslac7*-KO#3 and *Oslac7*-KO#4 plants as indicated. **d** Schematic drawing of the target site designed for knocking out *OsLAC28*. **e** Verification of two independent *Oslac28*-KO lines (*Oslac28*-KO#3 and *Oslac28*-KO#4) by PCR-based sequencing. **f** The alignment of OsLAC28 amino acid sequences encoded in Kitaake, *Oslac28*-KO#3 and *Oslac28*-KO#4 plants. **g** Schematic drawing of the target site designed for knocking out *OsLAC29*. **h** Verification of two independent *Oslac29*-KO lines (*Oslac29*-KO#1 and *Oslac29*-KO#2) by PCR-based sequencing. **i** The alignment of OsLAC29 amino acid sequences encoded in Kitaake, *Oslac29*-KO#1 and *Oslac29*-KO#2 plants. **j** Schematic drawing of the target site designed for knocking out *OsLAC29* in the genetic background of *Oslac7*-KO#3. **k** Verification of two independent *Oslac7/29*-KO lines (*Oslac7/29*-KO#3 and *Oslac7/29*-KO#8) by PCR-based sequencing. **l** Alignment of OsLAC29 amino acid sequences encoded in Kitaake, *Oslac7/29*-KO#3 and *Oslac7/29*-KO#8 plants.
