# Supplementary material for: Rice transcription factor bHLH25 confers resistance to multiple diseases by sensing H2O2
Source: Cell Res. 2025 Jan 14;35(3):205–19. doi: 10.1038/s41422-024-01058-4 (PMC11909244; doi:10.1038/s41422-024-01058-4)
Supplement: Supplementary file 3 — Fig. S3 [file 41422_2024_1058_MOESM3_ESM.pdf]

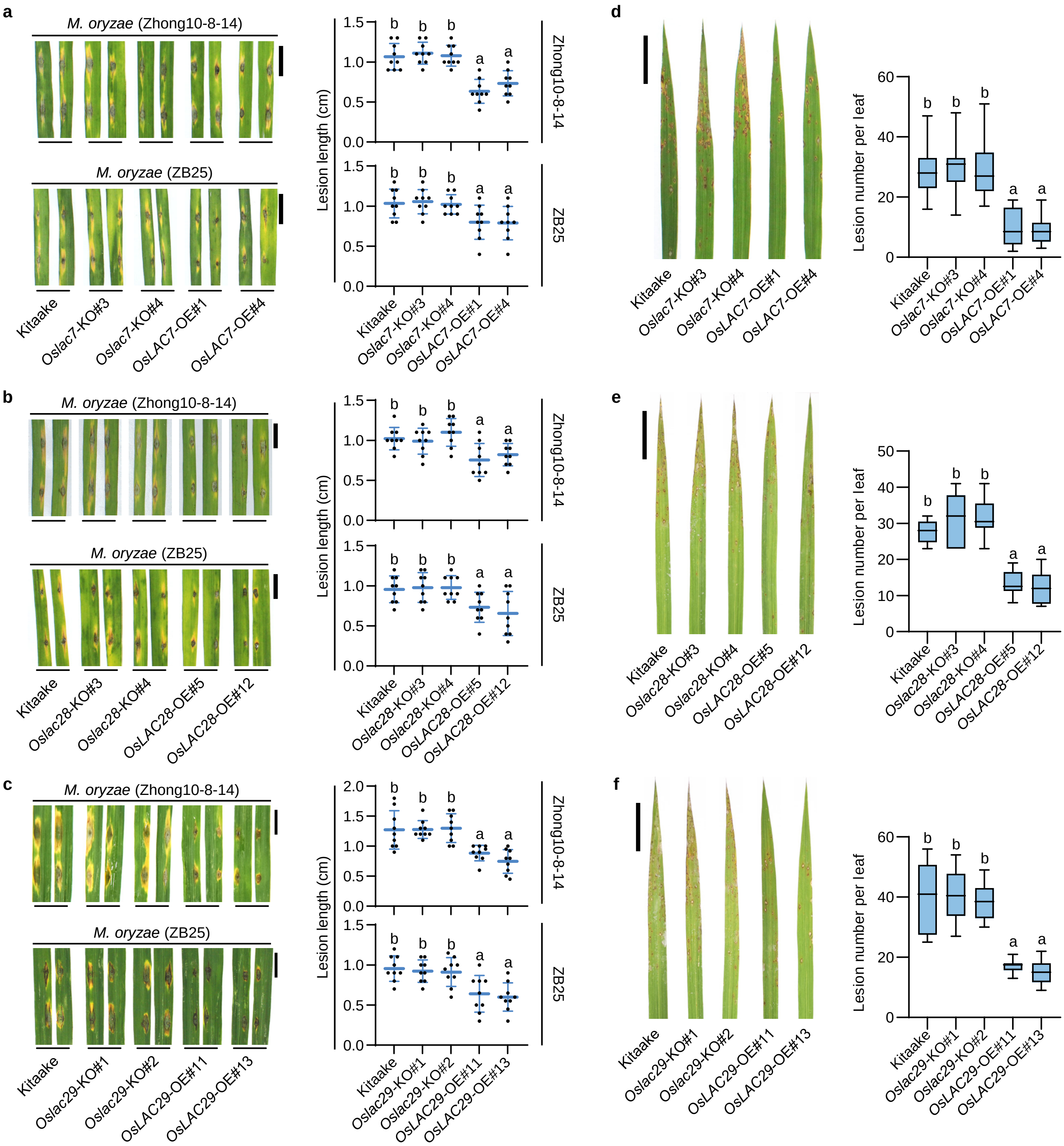

Supplementary information, Fig. S3 *OsLAC7/28/29* positively regulates plant resistance to *M. oryzae*. a-c Punch inoculation with *M. oryzae* Zhong10-8-14 (up panels) and ZB25 (down panels) in three-week-old plants of Kitaake, *Oslac7*-KO and *OsLAC7*-OE (**a**), of Kitaake, *Oslac28*-KO and *OsLAC28*-OE (**b**), and of Kitaake, *Oslac29*-KO and *OsLAC29*-OE (**c**). Photographs of representative lesions and lesion length (*n* = 9 lesions) in infected leaves at 7 dpi were shown. **d**-**f** Three-week-old plants of Kitaake, *Oslac7*-KO and *OsLAC7*-OE (**d**, *n* = 16 leaves), *Oslac28*-KO and *OsLAC28*-OE (**e**, *n* = 10 leaves), and *Oslac29*-KO and *OsLAC29*-OE (**f**, *n* = 10 leaves) were sprayed with *M. oryzae* Zhong10-8-14 for inoculation in rice field. Photographs of representative lesions and lesion numbers per leaf at 7 dpi are shown. Data are mean ± s.d. and analyzed by one-way ANOVA with LSD test. Scale bars are 1 cm (**a**-**c**) and 5 cm (**d**-**f**). Experiments were done with three biologically independent replications.
