# Supplementary material for: Rice transcription factor bHLH25 confers resistance to multiple diseases by sensing H2O2
Source: Cell Res. 2025 Jan 14;35(3):205–19. doi: 10.1038/s41422-024-01058-4 (PMC11909244; doi:10.1038/s41422-024-01058-4)
Supplement: Supplementary file 4 — Fig. S4 [file 41422_2024_1058_MOESM4_ESM.pdf]

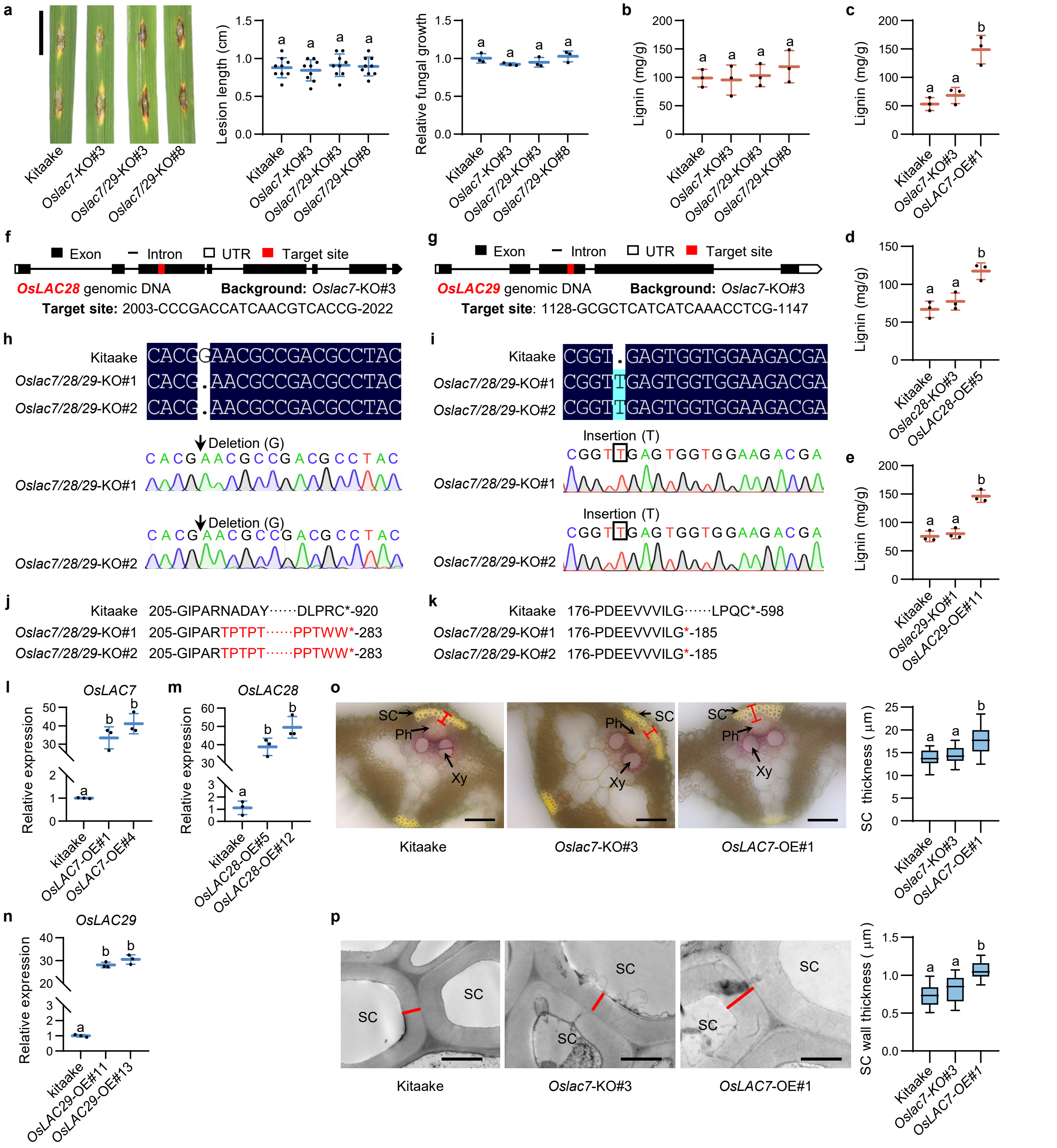

Supplementary information, Fig. S4 *OsLAC7/28/29* promotes lignin biosynthesis and sclerenchyma cell wall reinforcement. a Lesion length (*n* = 9 lesions) and fungal growth (*n* = 3 technical replicates) of three-week-old Kitaake, *Oslac7*-KO and *Oslac7/29*-KO plants at 7 dpi with Zhong10-8-14. **b** Lignin contents of four-week-old Kitaake and *Oslac7/29*-KO plants (*n* = 3 biological replicates). **c** Lignin contents of four-week-old Kitaake, *Oslac7*-KO and *OsLAC7*-OE plants (*n* = 3 biological replicates). **d** Lignin contents of four-week-old Kitaake, *Oslac28*-KO and *OsLAC28*-OE plants (*n* = 3 biological replicates). **e** Lignin contents of four-week-old Kitaake, *Oslac29*-KO and *OsLAC29*-OE plants (*n* = 3 biological replicates). **f-k** Identification of triple KO (*Oslac7/28/29*-KO) plants. Schematic drawing of two target sites designed for double KO of *OsLAC28* (**f**) and *OsLAC29* (**g**) in *Oslac7*-KO#3. Genomic DNA sequences of *OsLAC28* (**h**) and *OsLAC29* (**i**) in two independent *Oslac7/28/29*-KO lines (*Oslac7/28/29*-KO#1 and *Oslac7/28/29*-KO#2) were verified by PCR-based sequencing. The amino acid sequences encoded by *OsLAC28* (**j**) and *OsLAC29* (**k**) in Kitaake, *Oslac7/28/29*-KO#1 and *Oslac7/28/29*-KO#2 plants were aligned. **l** RNA levels of *OsLAC7* in three-week-old Kitaake and *OsLAC7*-OE plants (*n* = 3 technical replicates). **m** RNA levels of *OsLAC28* in three-week-old Kitaake and *OsLAC28*-OE plants (*n* = 3 technical replicates). **n** RNA levels of *OsLAC29* in three-week-old Kitaake and *OsLAC29*-OE plants (*n* = 3 technical replicates). **o** Histochemical staining of cross-sectioned leaves with phloroglucinol-HCl and thickness of sclerenchyma cells of four-week-old Kitaake, *Oslac7*-KO and *OsLAC7*-OE plants (*n* = 21 biological replicates). Ph, phloem; SC, sclerenchyma cells; Xy, xylem. Red markers: thickness of the SC layer. **p** Sclerenchyma cell wall thickness was quantified under a transmission electron microscope. Leaf sections are from four-week-old Kitaake, *Oslac7*-KO and *OsLAC7*-OE plants (*n* = 14 biological replicates). Data are mean ± s.d. and analyzed by one-way ANOVA with LSD test (**a-e**, **o**, **p**) or Dunnett's test (**l-n**). Scale bars are 1 cm (**a**), 50 μm (**o**) and 1 μm (**p**). Experiments were done with three biologically independent replications.
