# Supplementary material for: Rice transcription factor bHLH25 confers resistance to multiple diseases by sensing H2O2
Source: Cell Res. 2025 Jan 14;35(3):205–19. doi: 10.1038/s41422-024-01058-4 (PMC11909244; doi:10.1038/s41422-024-01058-4)
Supplement: Supplementary file 5 — Fig. S5 [file 41422_2024_1058_MOESM5_ESM.pdf]

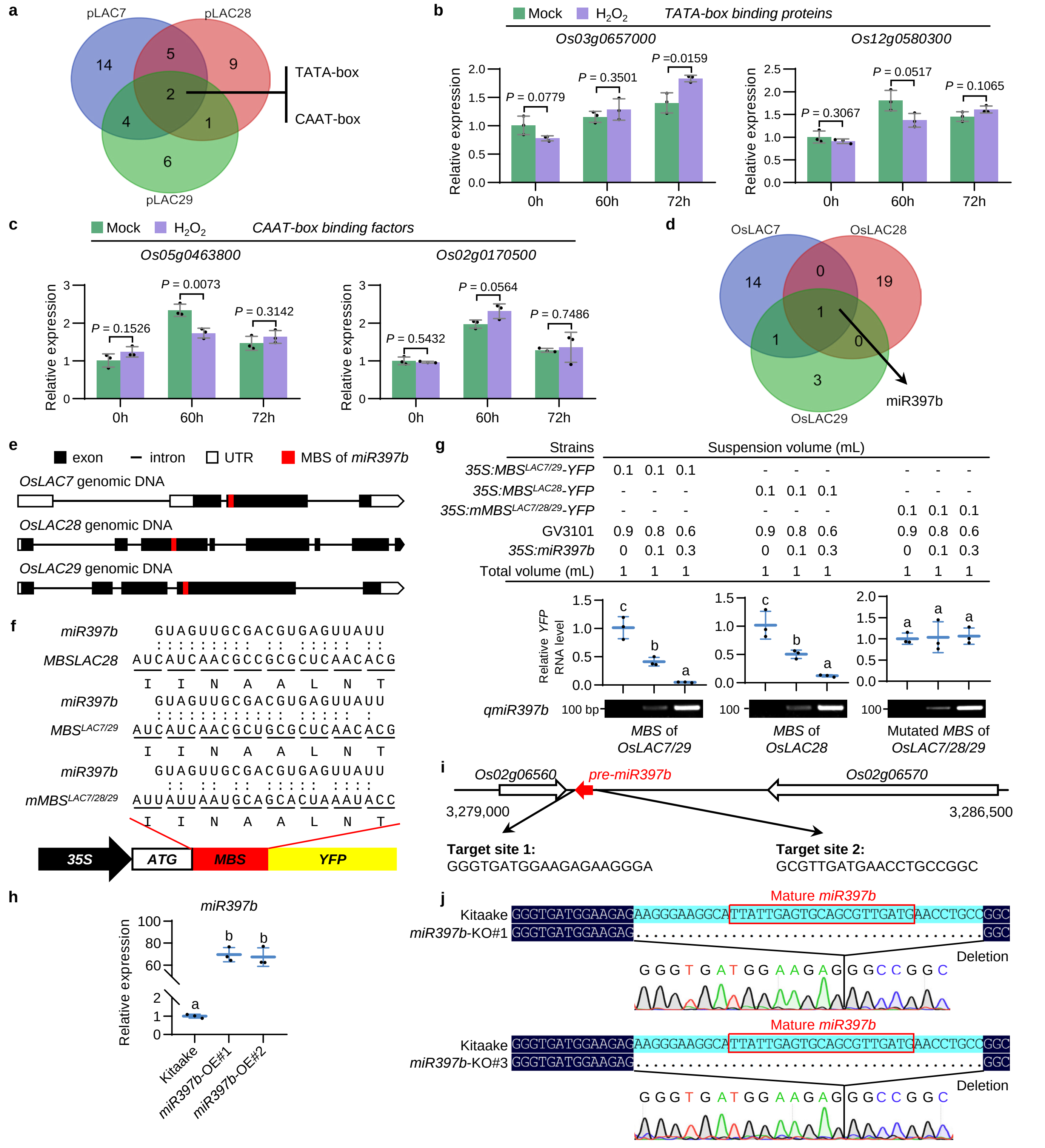

Supplementary information, Fig. S5 *miR397b* targets in *OsLAC7/28/29* and generation of *miR397b*-KO and *miR397b*-OE plants. a Pairwise comparisons of *cis*-elements in the promoters of *OsLAC7/28/29.* **b** RNA levels of *TATA-box-binding proteins* genes (*Os03g0657000* and *Os12g0580300*) in three-week-old Kitaake 0, 60 and 72 hpt with 1 mM H_2O_2 (mean ± s.d., *n* = 3 technical replicates). **c** RNA levels of *CAAT-box-binding factors* genes (*Os05g0463800* and *Os02g0170500*) in three-week-old Kitaake 0, 60 and 72 hpt with or without 1 mM H_2O_2 (mean ± s.d., *n* = 3 technical replicates). Mock treatment indicates without H_2O_2. **d** Pairwise comparisons for potential miRNA target sites in the mRNAs of *OsLAC7/28/29.* **e** Schematic drawing of the miRNA binding site (MBS) (in red box) of *miR397b* in the mRNAs of *OsLAC7/28/29* genes. **f** Alignments of *miR397b* with the MBS and the mutated MBS (mMBS) of *miR397b* in the mRNAs of *OsLAC7/28/29*. **g** Transactivation assays to assess *miR397b* function on *OsLAC7/28/29* expression*.* The *YFP* RNA levels were determined by RT-qPCR analysis (*n* = 3 technical replicates). The *miR397b* level was determined by semi-quantitative PCR. **h** Expression levels of *miR397b* in three-week-old Kitaake and *miR397b*-OE plants (*n* = 3 technical replicates). **i** Schematic drawing of two target sites designed for knocking out the precursor of *miR397b* (in red arrow) by the CRISPR/Cas9 system. **j** Verification of two independent *miR397b*-KO lines (*miR397b*-KO#1 and *miR397b*-KO#3) by PCR-based sequencing. Both mutant lines show a deletion of the 40 bp fragment containing mature *miR397b* (highlighted in red boxes). Data are mean ± s.d. and analyzed by two-tailed Student's *t*-test (**b**, **c**) and one-way ANOVA with LSD test (**g**, **h**). Experiments were done with three biologically independent replications.
