# Supplementary material for: Rice transcription factor bHLH25 confers resistance to multiple diseases by sensing H2O2
Source: Cell Res. 2025 Jan 14;35(3):205–19. doi: 10.1038/s41422-024-01058-4 (PMC11909244; doi:10.1038/s41422-024-01058-4)
Supplement: Supplementary file 6 — Fig. S6 [file 41422_2024_1058_MOESM6_ESM.pdf]

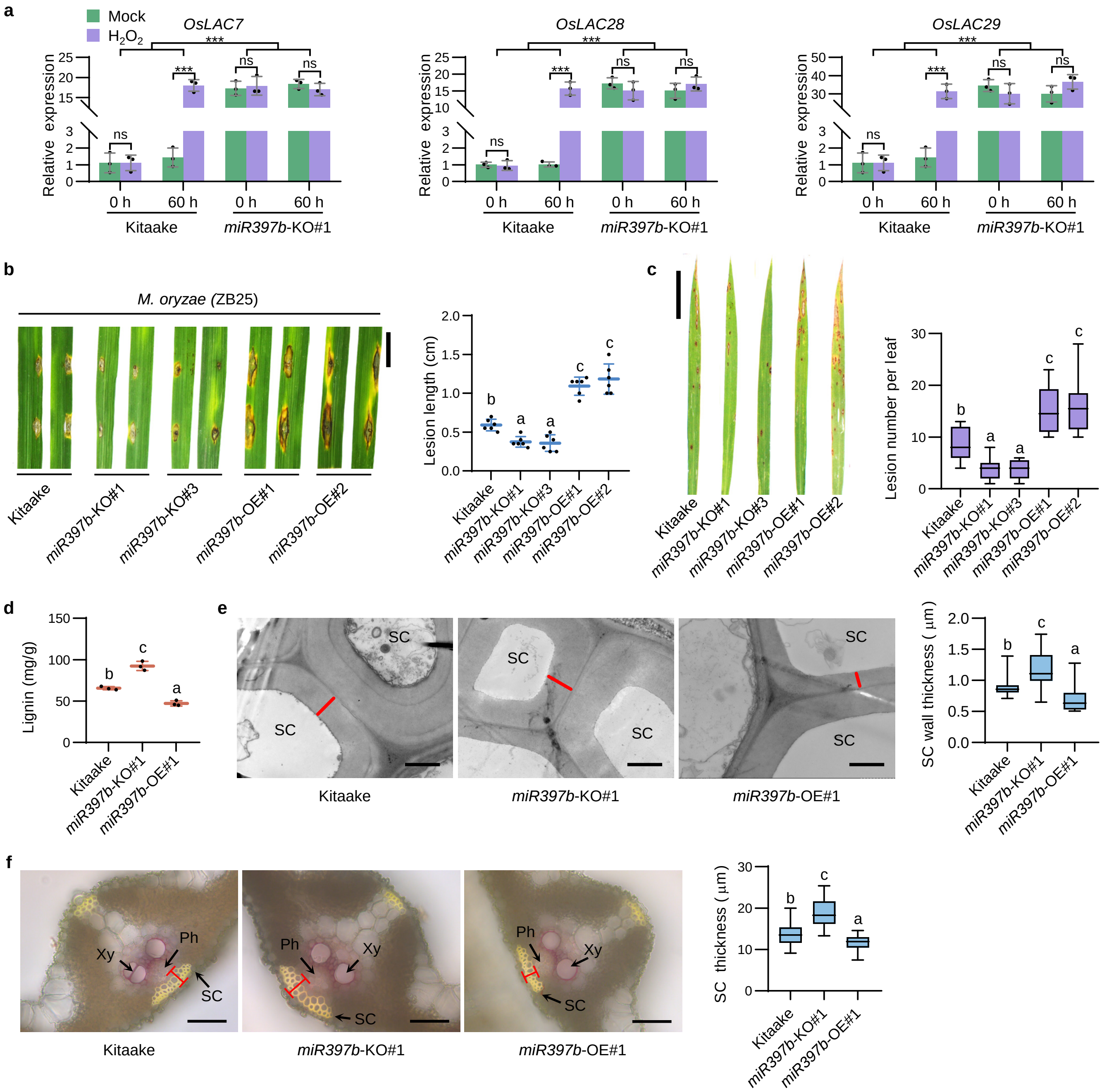

Supplementary information, Fig. S6 *miR397b* regulates *OsLAC7/28/29*-mediated cell wall reinforcement and disease resistance in rice. a RNA levels of *OsLAC7/28/29* in the leaves of Kitaake and *miR397b*-KO plants after H_2O_2 treatment (n = 3 technical replicates). **b** Lesion length (n = 6 lesions) of three-week-old Kitaake, *miR397b*-KO and *miR397b*-OE plants at 7 dpi with *M. oryzae* ZB25. **c** Three-week-old Kitaake, *miR397b*-KO and *miR397b*-OE plants were sprayed with *M. oryzae* Zhong10-8-14 for inoculation in field. Photographs of representative lesions and lesion numbers per leaf ($n \geq 10$ leaves) at 7 dpi are shown. **d** Lignin contents in four-week-old Kitaake, *miR397b*-KO and *miR397b*-OE plants (n = 3 biological replicates). **e** Sclerenchyma cell walls observed under a transmission electron microscope. Sclerenchyma cell wall thickness is quantified (n = 18 biological replicates). Leaf sections are from four-week-old Kitaake, *miR397b*-KO and *miR397b*-OE plants. **f** Histochemical staining of cross-sectioned leaves with phloroglucinol-HCl and thickness of sclerenchyma cells (n = 21 biological replicates) in four-week-old Kitaake, *miR397b*-KO and *miR397b*-OE plants. Scale bars are 1 cm (**b**), 5 cm (**c**), 1 μm (**e**) and 50 μm (**f**). Data are mean ± s.d. and analyzed by two-way ANOVA with Tukey's test at ***$P < 0.001$; ns, not significant (**a**) and one-way ANOVA with LSD test (**b**-**f**). Experiments were done with three biologically independent replications.
