# Supplementary material for: Rice transcription factor bHLH25 confers resistance to multiple diseases by sensing H2O2
Source: Cell Res. 2025 Jan 14;35(3):205–19. doi: 10.1038/s41422-024-01058-4 (PMC11909244; doi:10.1038/s41422-024-01058-4)
Supplement: Supplementary file 7 — Fig. S7 [file 41422_2024_1058_MOESM7_ESM.pdf]

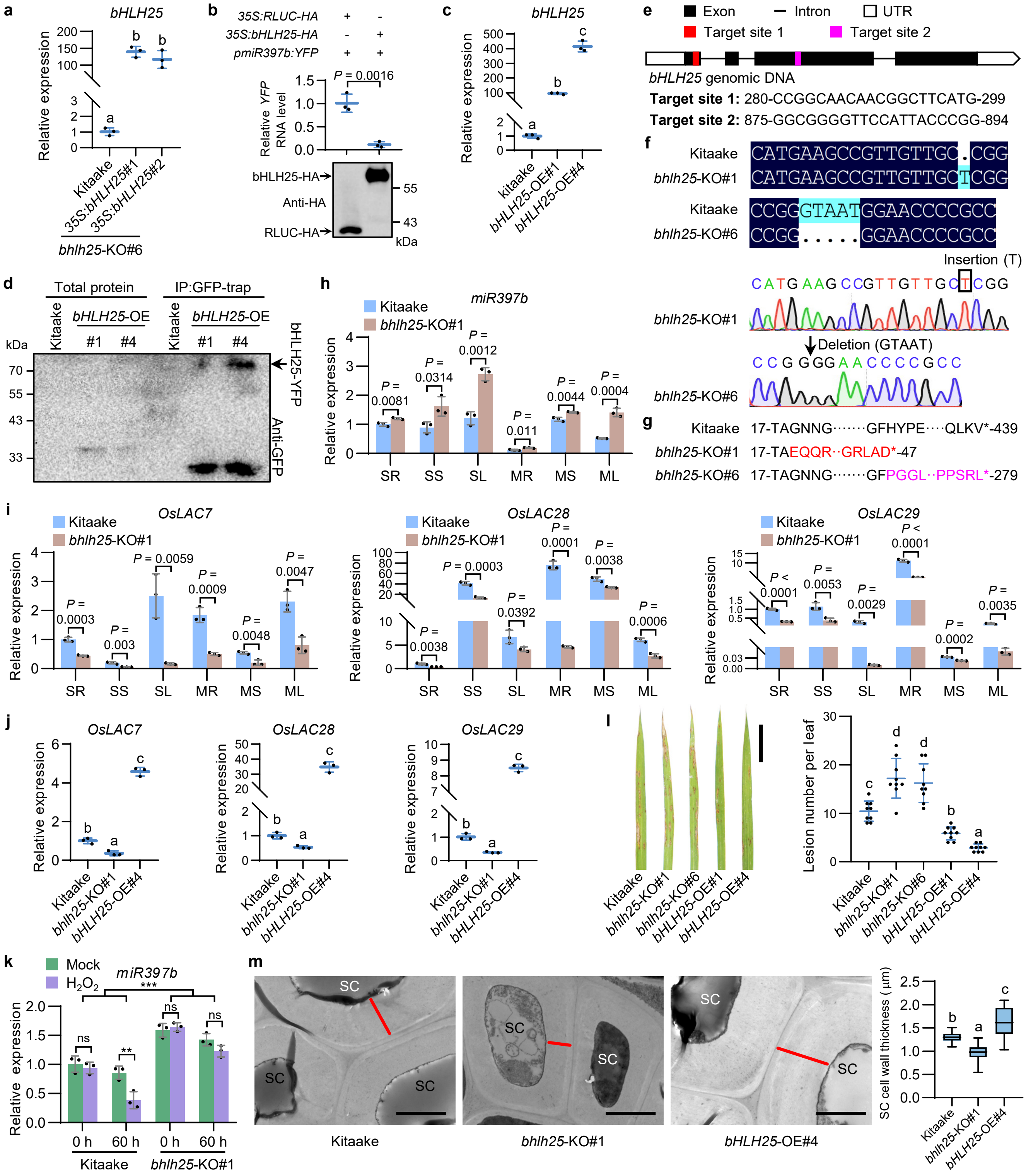

Supplementary information, Fig. S7 *bHLH25* promotes lignin biosynthesis and disease resistance in rice. a RNA levels of *bHLH25* in three-week-old Kitaake plants and plants overexpressing *bHLH25* in *bhlh25*-KO#6 background (n = 3 technical replicates). **b** Transactivation assay shows that bHLH25 represses *pmiR397b* activity in *N. benthaminana*. *35S:RLUC-HA* was used as a negative control. *YFP* RNA levels were measured by RT-qPCR analysis (n = 3 technical replicates). RLUC-HA and bHLH25-HA were detected by immunoblot analysis. **c** RNA levels of *bHLH25* in three-week-old Kitaake and *bHLH25*-OE plants (n = 3 technical replicates). **d** bHLH25 accumulation in four-week-old *bHLH25*-OE plants was assayed by immunoblotting. The arrow indicates immunoprecipitated bHLH25-YFP protein. **e** Schematic drawing of two independent target sites designed for knocking out the *bHLH25* gene by the CRISPR/Cas9 system. **f** Verification of two independent *bhlh25*-KO lines by PCR-based sequencing. **g** The amino acid sequence alignment of bHLH25 proteins of Kitaake, *bhlh25*-KO#1 and *bhlh25*-KO#6 plants. Each mutation leads to a frameshift and premature termination in the bHLH25 protein. **h**, **i** RNA levels of *miR397b* (**h**) and *OsLAC7/28/29* (**i**) in roots, stems and leaves of three-week-old seedlings and three-month-old mature plants of Kitaake and *bhlh25*-KO (n = 3 technical replicates). SR and MR : roots of seedling and mature plants. SS and MS: stems of seedling and mature plants. SL and ML: leaves of seedling and mature plants. **j** RNA levels of *OsLAC7/28/29* in three-week-old Kitaake, *bhlh25*-KO and *bHLH25*-OE plants (n = 3 technical replicates). **k** RNA levels of *miR397b* in the leaves of Kitaake and *bhlh25*-KO plants after H_2O_2 treatment (n = 3 technical replicates). **l** Lesion numbers per leaf (n = 9 leaves) of three-week-old Kitaake, *bhlh25*-KO and *bHLH25*-OE sprayed with Zhong10-8-14 at 7 dpi in field. **m** Sclerenchyma cell wall thickness (n = 19 biological replicates) of leaf sections from four-week-old Kitaake, *bhlh25*-KO and *bHLH25*-OE plants. Data are mean ± s.d. and analyzed by one-way ANOVA with Dunnett's test (**a**, **c**, **j**) or LSD (**l**, **m**), two-tailed Student's *t*-test (**b**, **h**, **i**) and two-way ANOVA with Tukey's test at $^{**}P < 0.01$, $^{***}P < 0.001$; ns, not significant (**k**). Scale bars are 5 cm (**l**) and 1 μm (**m**). Experiments were done with three biologically independent replications.
