# Supplementary material for: Rice transcription factor bHLH25 confers resistance to multiple diseases by sensing H2O2
Source: Cell Res. 2025 Jan 14;35(3):205–19. doi: 10.1038/s41422-024-01058-4 (PMC11909244; doi:10.1038/s41422-024-01058-4)
Supplement: Supplementary file 8 — Fig. S8 [file 41422_2024_1058_MOESM8_ESM.pdf]

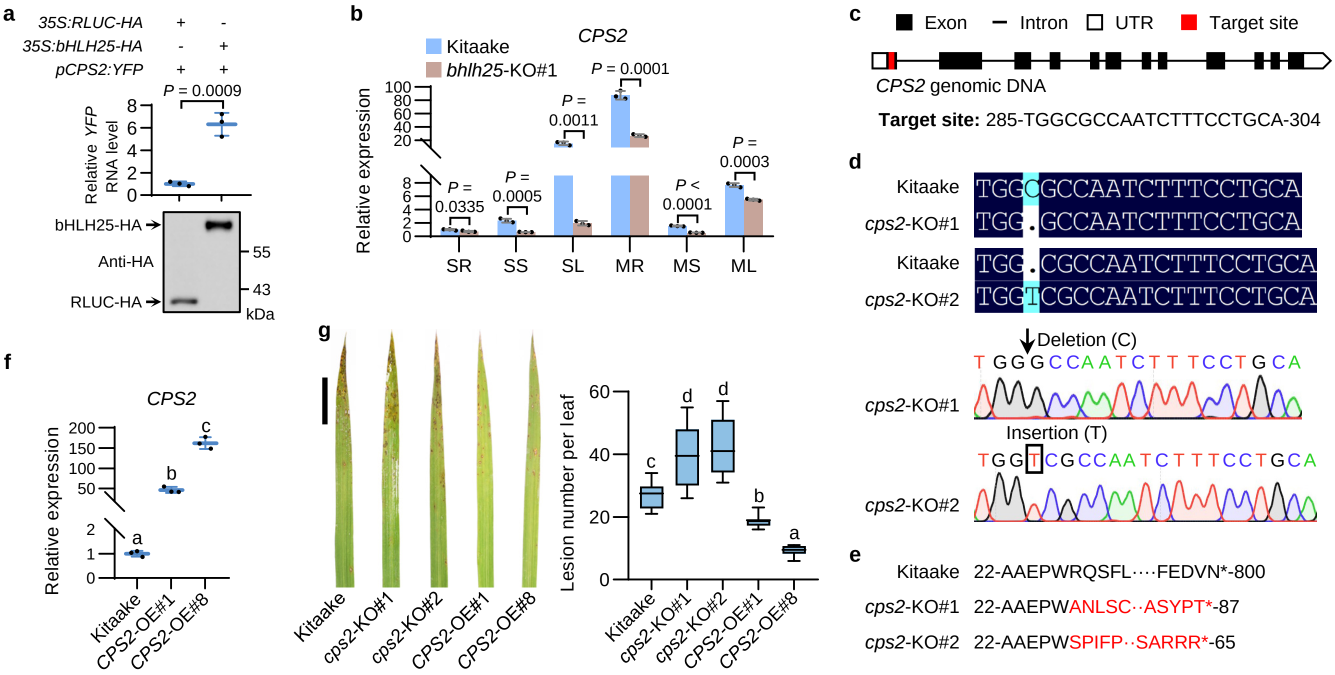

Supplementary information, Fig. S8 bHLH25 promotes *CPS2* expression to enhance rice disease resistance. a Transactivation assay shows that bHLH25 increases *pCPS2* activity. *35S:RLUC-HA* was used as a negative control. *YFP* RNA levels were measured by RT-qPCR analysis (n = 3 technical replicates). RLUC-HA and bHLH25-HA were detected by immunoblot analysis. Data were collected from the same *N. benthamiana* leaf. **b** RNA levels of *CPS2* in roots, stems and leaves of three-week-old seedlings and three-month-old mature plants of Kitaake and *bhlh25*-KO (n = 3 technical replicates). **c** Schematic drawing of the target site designed for knocking out *CPS2* in Kitaake plants. **d** Verification of two independent *cps2*-KO lines (*cps2*-KO#1 and *cps2*-KO#2) by PCR-based sequencing. **e** Alignment of CPS2 amino acid sequences encoded in Kitaake, *cps2*-KO#1 and *cps2*-KO#2 plants as indicated. **f** RNA levels of *CPS2* in three-week-old Kitaake and *CPS2*-OE plants (mean ± s.d., n = 3 technical replicates). **g** Three-week-old Kitaake, *cps2*-KO and *CPS2*-OE plants were sprayed with Zhong10-8-14 for inoculation in field. Photographs of representative lesions and lesion numbers per leaf (mean ± s.d., n = 12 leaves) at 7 dpi are shown. Scale bar, 5 cm. Data are mean ± s.d. and analyzed by two-tailed Student's t-test (**a**, **b**) and one-way ANOVA with LSD test (**f**, **g**). Experiments were done with three biologically independent replications.
