# Supplementary material for: Rice transcription factor bHLH25 confers resistance to multiple diseases by sensing H2O2
Source: Cell Res. 2025 Jan 14;35(3):205–19. doi: 10.1038/s41422-024-01058-4 (PMC11909244; doi:10.1038/s41422-024-01058-4)
Supplement: Supplementary file 9 — Fig. S9 [file 41422_2024_1058_MOESM9_ESM.pdf]

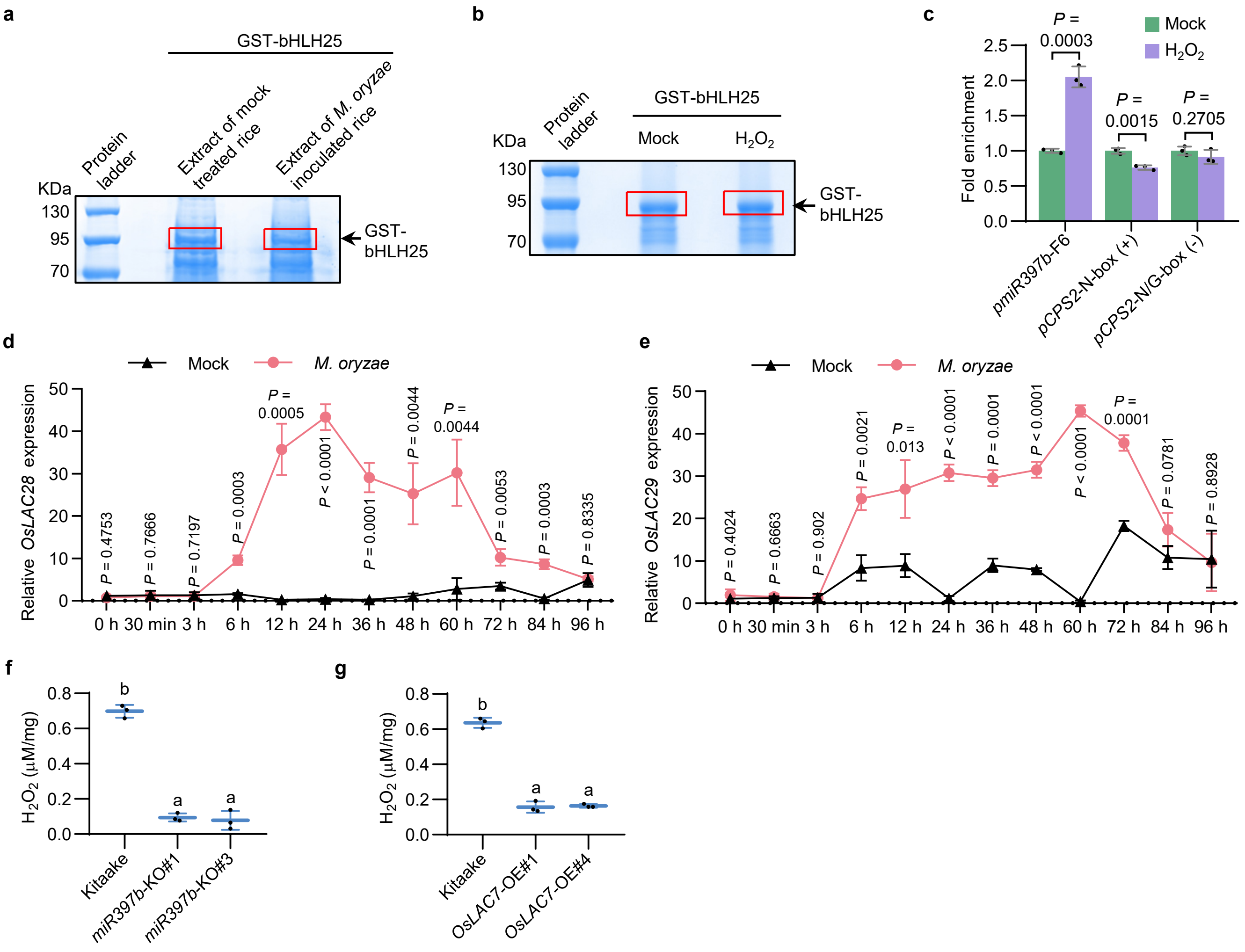

Supplementary information, Fig. S9 H_2O_2 controls the ability of bHLH25 to induce biosynthesis of lignin and phytoalexin in rice by *M. oryzae* infection. a Coomassie brilliant blue (CBB) staining of GST-bHLH25 protein incubated with extracts of Kitaake leaves pre-treated with *M. oryzae* or with mock treatments. **b** CBB staining of GST-bHLH25 protein directly treated with mock or H_2O_2. Red boxes show the bands cut out for mass spectrometry analysis (**a**, **b**). **c** H_2O_2 alters the DNA-binding specificity of bHLH25 in DAP-qPCR assay (n = 3 technical replicates). GST-bHLH25 was pre-treated with or without H_2O_2. The *pmiR397b*-F6 contains G-box-like-2 motif. The *pCPS2*-N-box (+) contains N-box-like motif. The *pCPS2*-N/G-box (-) containing no N-box-like or G-box-like motif indicates negative control. **d**, **e** Levels of *OsLAC28* mRNA (**d**) and *OsLAC29* mRNA (**e**) in three-week-old Kitaake leaves at 0-96 hpi with or without Zhong10-8-14 (n = 3 technical replicates). **f** Endogenous H_2O_2 contents in three-week-old Kitaake and *miR397b*-KO plants (n = 3 biological replicates). **g** Endogenous H_2O_2 contents in three-week-old Kitaake and *OsLAC7*-OE plants (n = 3 biological replicates). Data are mean ± s.d. and analyzed by two-tailed Student's *t*-test (**c-e**) and one-way ANOVA with LSD test (**f**, **g**). Experiments were done with three biologically independent replications.
