# Supplementary material for: Rice transcription factor bHLH25 confers resistance to multiple diseases by sensing H2O2
Source: Cell Res. 2025 Jan 14;35(3):205–19. doi: 10.1038/s41422-024-01058-4 (PMC11909244; doi:10.1038/s41422-024-01058-4)
Supplement: Supplementary file 10 — Fig. S10 [file 41422_2024_1058_MOESM10_ESM.pdf]

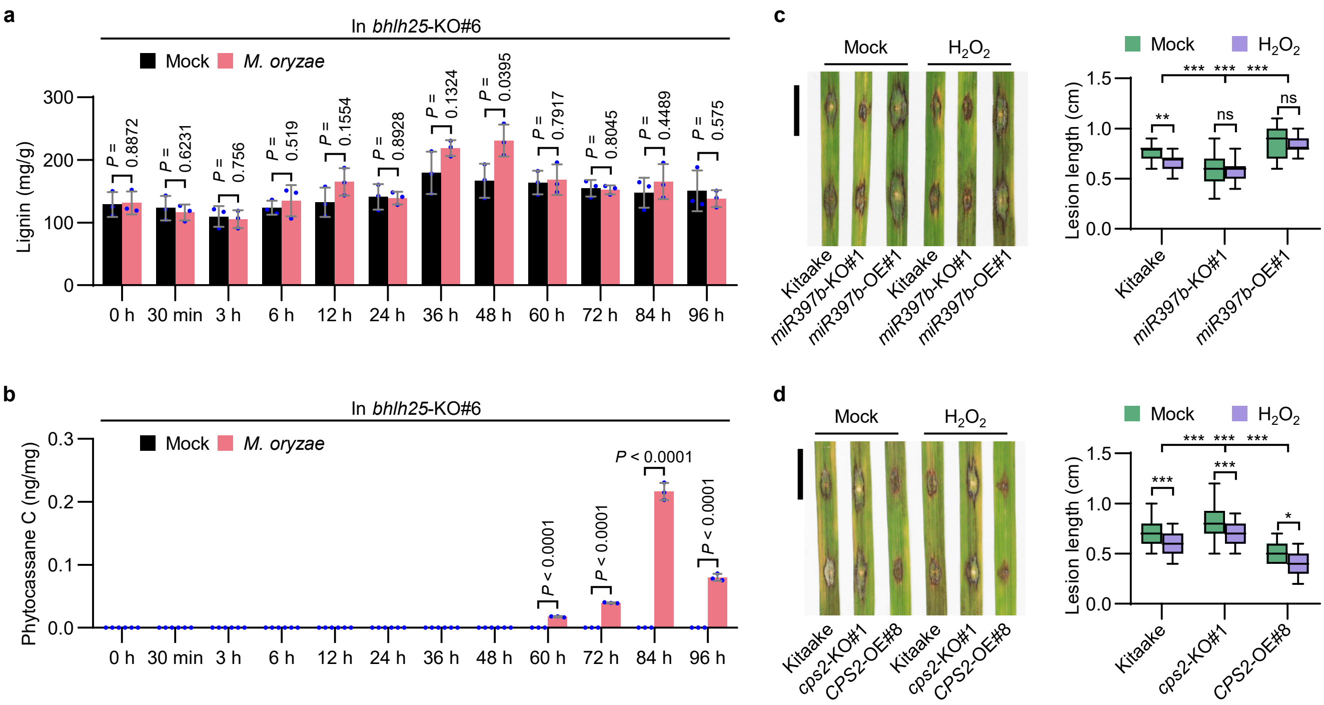

Supplementary information, Fig. S10 Induction of lignin, phytoalexin and disease resistance in rice by *M. oryzae* infection depends on *bHLH25*. a, **b** lignin content (**a**) and phytocassane C content (**b**) were measured in leaves of three-week-old *bhlh25*-KO#6 plants at 0-96 hpi with or without *M. oryzae* Zhong10-8-14 (n = 3 biological replicates). **c**, **d** Three-week-old *miR397b*-KO/OE (**c**), *CPS2*-KO/OE (**d**) and Kitaake plants were pre-treated with or without 1 mM H_2O_2 on roots for 72 h, then their leaves were inoculated with Zhong10-8-14. Representative lesions and lesion length (n = 30 lesions) at 7 dpi are shown. Data are mean ± s.d. and analyzed by two-tailed Student's t-test (**a**, **b**) and two-way ANOVA with Tukey's test at $^*P < 0.05$, $^{**}P < 0.01$, $^{***}P < 0.001$; ns, not significant (**c**, **d**). Scale bar, 1 cm (**c**, **d**). Experiments were done with three biologically independent replications.
