# Supplementary material for: Rice transcription factor bHLH25 confers resistance to multiple diseases by sensing H2O2
Source: Cell Res. 2025 Jan 14;35(3):205–19. doi: 10.1038/s41422-024-01058-4 (PMC11909244; doi:10.1038/s41422-024-01058-4)
Supplement: Supplementary file 12 — Fig. S12 [file 41422_2024_1058_MOESM12_ESM.pdf]

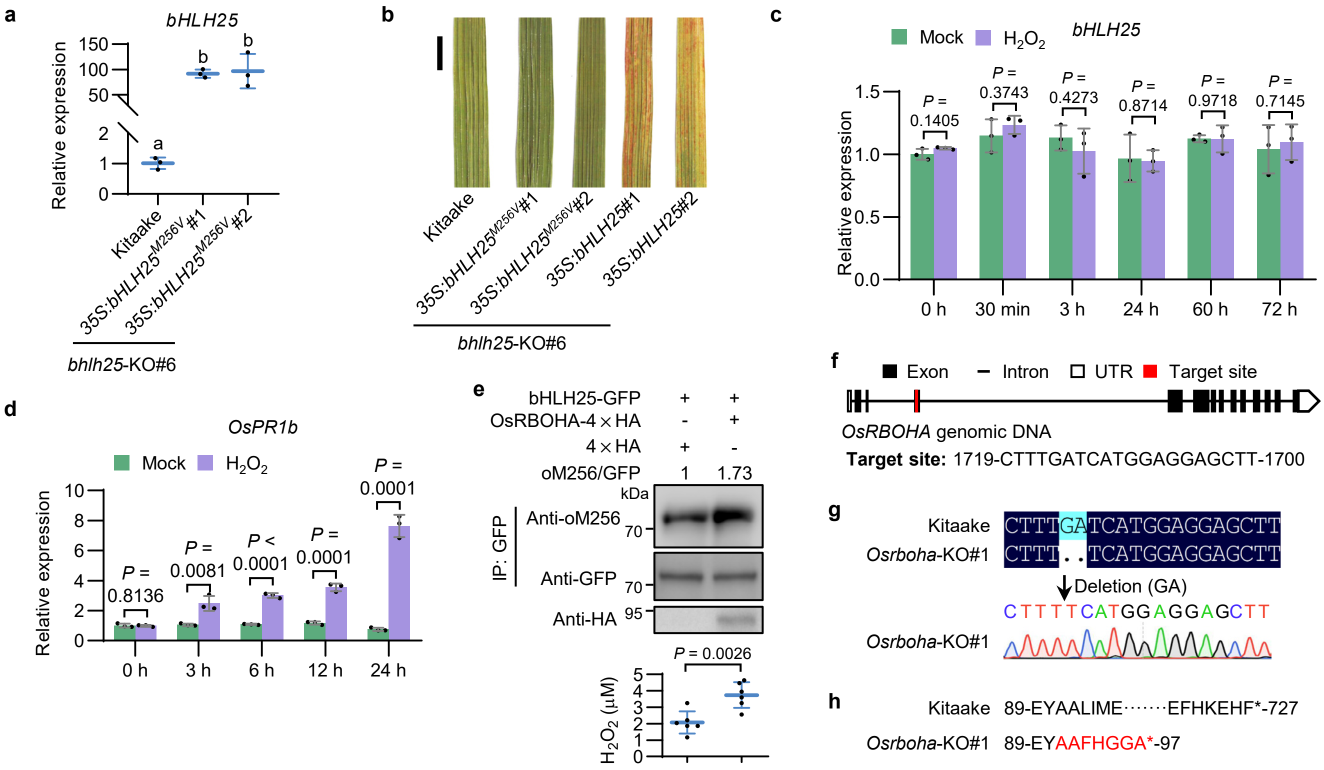

Supplementary information, Fig. S12 M256 of bHLH25 directly senses H_2O_2 in vivo. a RT-qPCR analysis shows bHLH25 RNA levels in three-week-old plants overexpressing bHLH25^{M256V}-YFP in *bhlh25*-KO#6 (*n* = 3 technical replicates). **b** Representative leaves of plants overexpressing bHLH25-YFP or bHLH25^{M256V}-YFP in *bhlh25*-KO#6 at the tillering stage. Scale bar, 1 cm. **c** RNA levels of bHLH25 in three-week-old Kitaake roots treated with or without 1 mM H_2O_2 (*n* = 3 technical replicates). **d** RNA levels of *OsPR1b* in chipped rice leaves after direct treatment with H_2O_2 (*n* = 3 technical replicates). **e** In vivo oxidation of M256 in bHLH25 in the presence of *OsRBOHA*. Protoplasts from two-week-old Kitaake plants were transformed to co-express bHLH25-GFP with OsRBOHA-4×HA or 4×HA (negative control). Endogenous H_2O_2 levels in protoplasts were measured (*n* = 6 biological replicates). **f** Schematic drawing of the target site designed for knocking out *OsRBOHA* in wild-type Kitaake plants. **g** Verification *Osrboha*-KO#1 by PCR-based sequencing. **h** The alignment of OsRBOHA amino acid sequences encoded in Kitaake and *Osrboha*-KO#1 plants. Data are mean ± s.d. and analyzed by one-way ANOVA with LSD test (**a**) and two-tailed Student's *t*-test (**c**-**e**). Experiments were done with three biologically independent replications.
