# Supplementary material for: Rice transcription factor bHLH25 confers resistance to multiple diseases by sensing H2O2
Source: Cell Res. 2025 Jan 14;35(3):205–19. doi: 10.1038/s41422-024-01058-4 (PMC11909244; doi:10.1038/s41422-024-01058-4)
Supplement: Supplementary file 13 — Fig. S13 [file 41422_2024_1058_MOESM13_ESM.pdf]

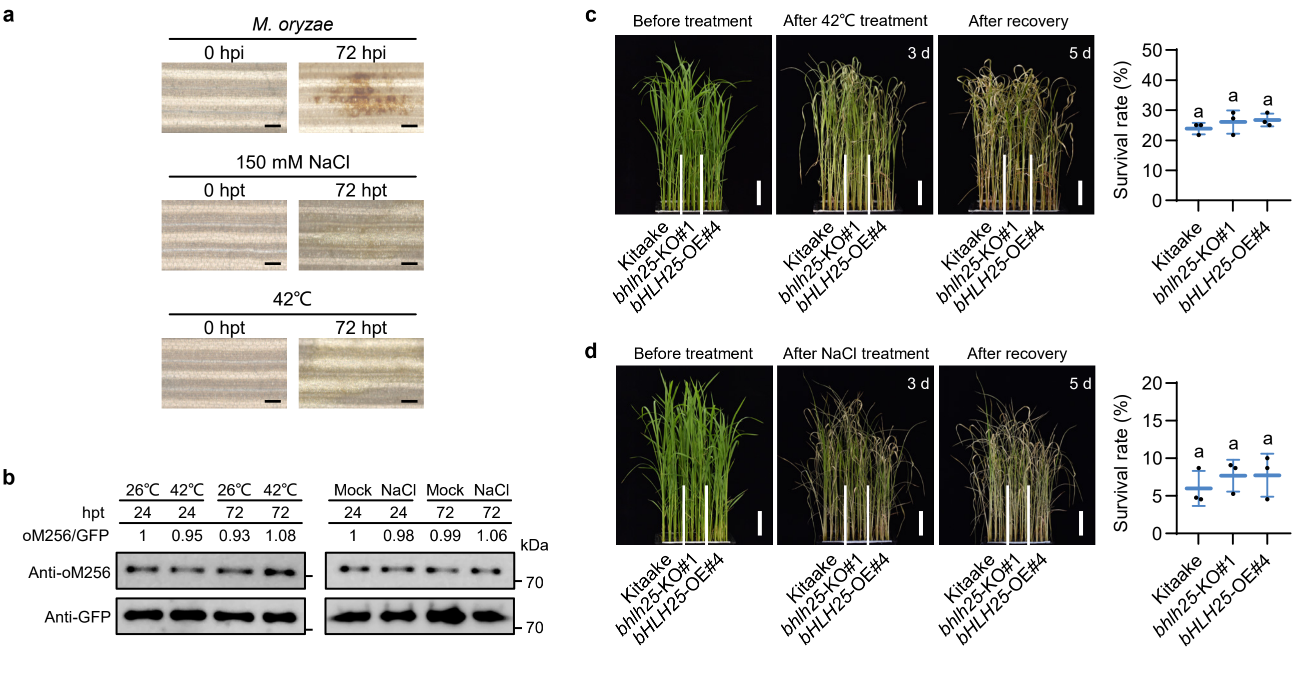

Supplementary information, Fig. S13 bHLH25 has no obvious role in plant abiotic stress responses. a 3,3-diaminobenzidine (DAB) staining shows H_2O_2 accumulation in leaves of rice plants at 72 hpt with *M. oryzae* Zhong10-8-14, 150 mM NaCl, or 42℃. **b** The in vivo oxidation level of bHLH25 in leaves of rice plants at 24 and 72 hpt with 150 mM NaCl or with 42℃. **c**, **d** Representative plants and survival rates (n = 3 biological replicates) of three-week-old Kitaake, *bhlh25*-KO, and *bHLH25*-OE plants before and after 42℃ high temperature treatment (**c**) or 150 mM NaCl (**d**) for three days in a growth chamber, followed by recovery at normal condition for five days. Data are mean ± s.d. and analyzed by one-way ANOVA with LSD test. Scale bars, 0.1 mm (**a**) and 5 cm (**c**).
