# Supplementary material for: Rice transcription factor bHLH25 confers resistance to multiple diseases by sensing H2O2
Source: Cell Res. 2025 Jan 14;35(3):205–19. doi: 10.1038/s41422-024-01058-4 (PMC11909244; doi:10.1038/s41422-024-01058-4)
Supplement: Supplementary file 14 — Fig. S14 [file 41422_2024_1058_MOESM14_ESM.pdf]

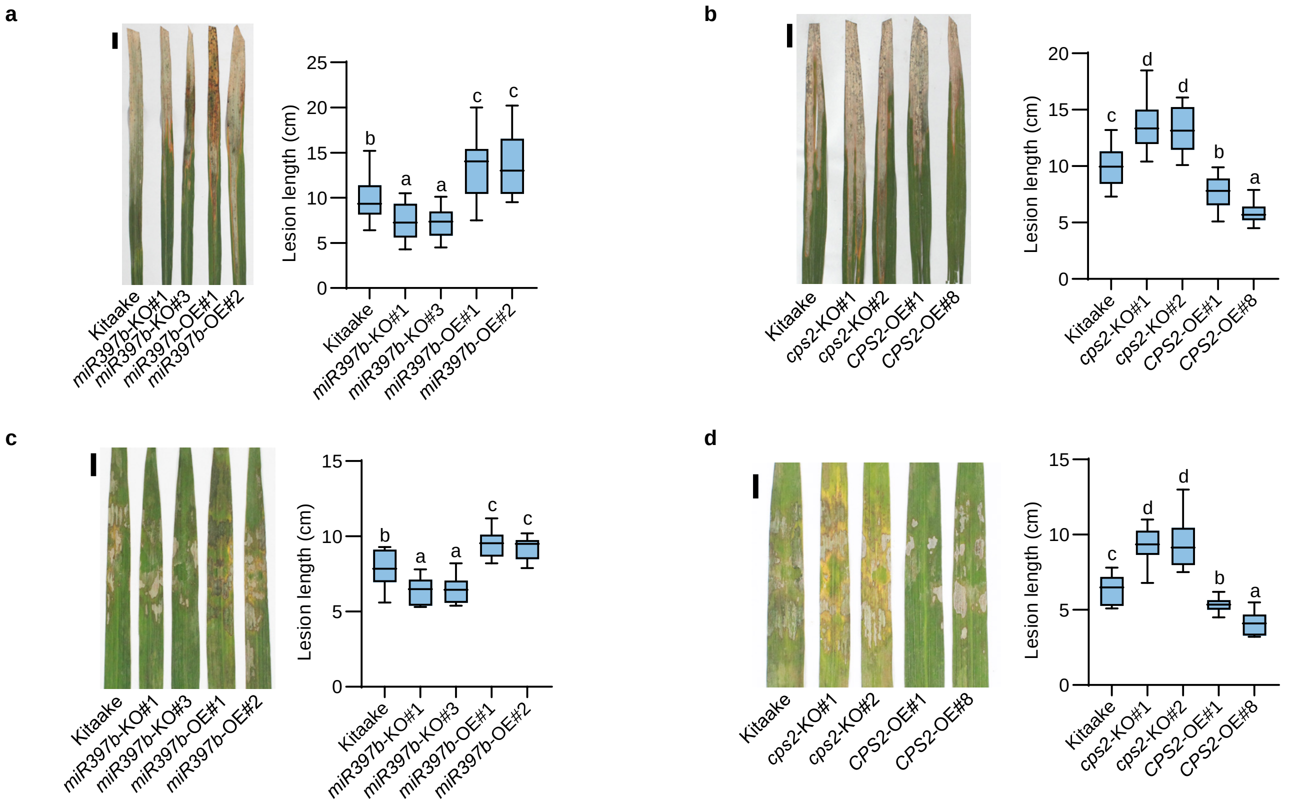

Supplementary information, Fig. S14 *miR397b* and *CPS2* regulate plant resistance to multiple diseases. a Photographs of representative lesions and lesion length (*n* = 20 lesions) of tillering-stage Kitaake, *miR397b*-KO and *miR397b*-OE plants at 14 dpi with *Xoo* PXO99A. **b** Photographs of representative lesions and lesion length (*n* = 20 lesions) of tillering-stage Kitaake, *cps2*-KO and *CPS2*-OE plants at 14 dpi with *Xoo* PXO99A. **c** Photographs of representative lesions and lesion length (*n* = 10 lesions) of tillering-stage Kitaake, *miR397b*-KO and *miR397b*-OE plants at 2 dpi with *R. solani* AG-1-IA. **d** Photographs of representative lesions and lesion length (*n* = 10 lesions) of tillering-stage Kitaake, *cps2*-KO and *CPS2*-OE plants at 2 dpi with *R. solani* AG-1-IA. Scale bar, 1 cm. Data are mean ± s.d. and analyzed by one-way ANOVA with LSD test. Experiments were done with three biologically independent replications.
