# Supplementary material for: Rice transcription factor bHLH25 confers resistance to multiple diseases by sensing H2O2
Source: Cell Res. 2025 Jan 14;35(3):205–19. doi: 10.1038/s41422-024-01058-4 (PMC11909244; doi:10.1038/s41422-024-01058-4)
Supplement: Supplementary file 15 — Fig. S15 [file 41422_2024_1058_MOESM15_ESM.pdf]

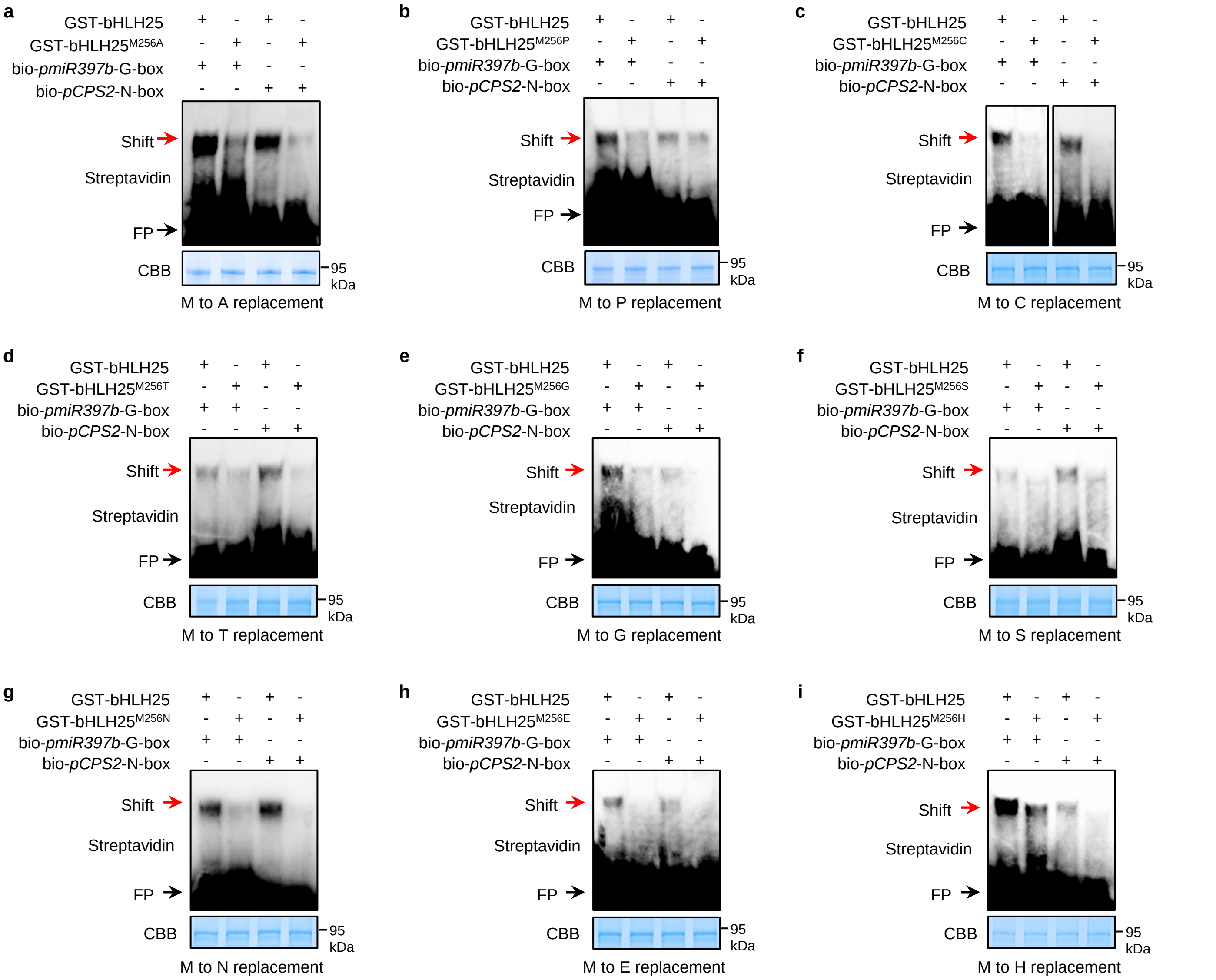

Supplementary information, Fig. S15 M256 residue is required for the DNA binding ability of bHLH25. a-i EMSA for determining the effect of M256 replacement by hydrophobic amino acids of A and P (**a**, **b**), hydrophilic amino acids of C, T, G, S and N (**c**-**g**), acidic amino acids of E (**h**) and basic amino acids of H (**i**) on the DNA binding ability of bHLH25 to *pmiR397b* and *pCPS2*. The red arrow indicates biotin labelled probes bound to GST-bHLH25 or its M256 mutant proteins. FP: free probes. The proteins levels of GST-bHLH25 and its M256 mutant proteins were quantified to an equal amount and shown in CBB staining.
