# Supplementary material for: Rice transcription factor bHLH25 confers resistance to multiple diseases by sensing H2O2
Source: Cell Res. 2025 Jan 14;35(3):205–19. doi: 10.1038/s41422-024-01058-4 (PMC11909244; doi:10.1038/s41422-024-01058-4)
Supplement: Supplementary file 11 — Fig. S11 [file 41422_2024_1058_MOESM11_ESM.pdf]

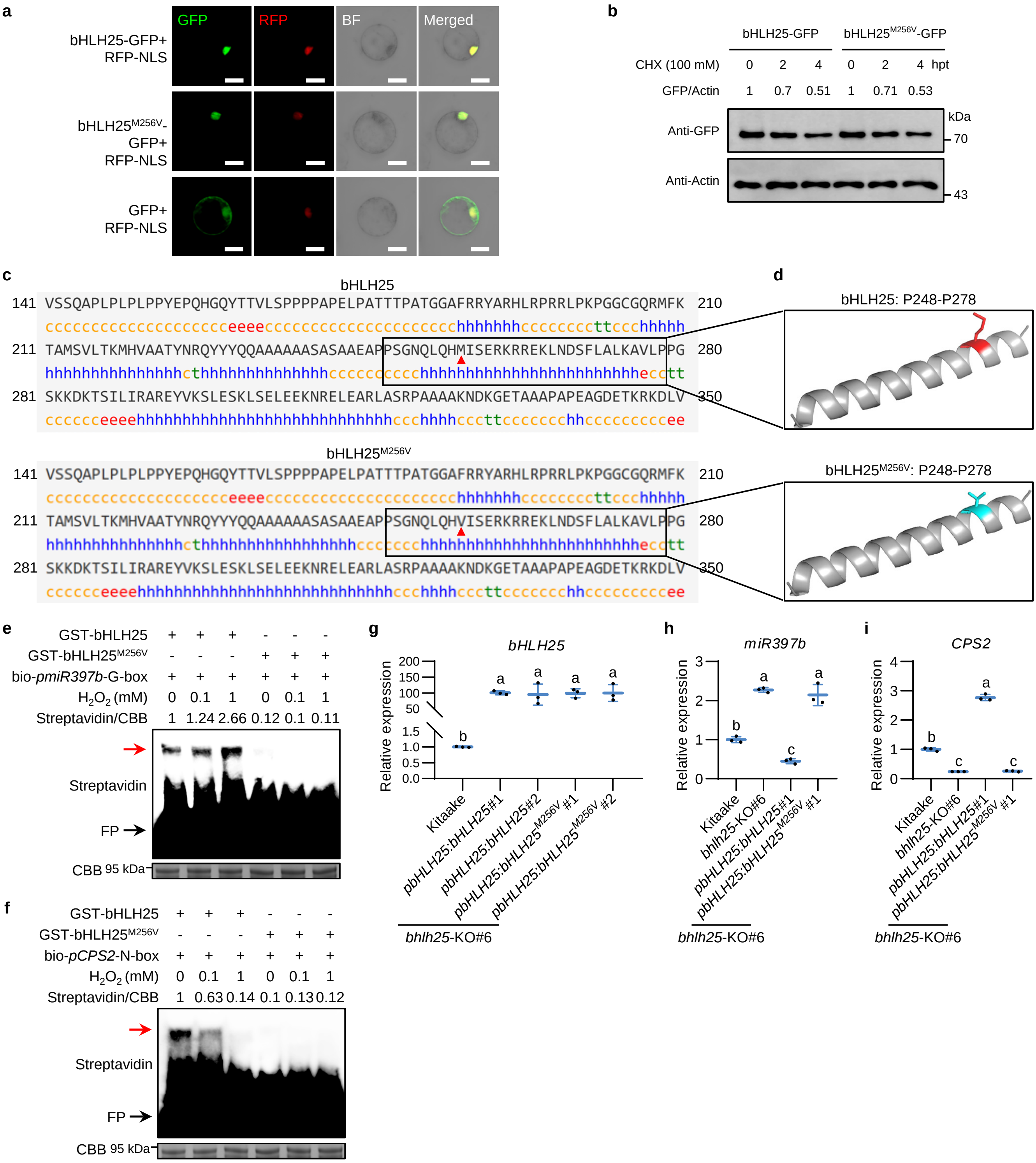

Supplementary information, Fig. S11 M256 is essential for the DNA binding ability of bHLH25. a Subcellular localization of bHLH25 and bHLH25^{M256V} proteins in rice protoplasts from two-week-old Kitaake plants. Scale bar, 10 μm. **b** The determination of the protein stability of bHLH25 and bHLH25^{M256V} *in vivo*. Cycloheximide (CHX) were used as the protein biosynthesis inhibitor. **c** Secondary structure prediction for bHLH25 and bHLH25^{M256V}. The black box indicates the alpha helix structure. The red triangles indicate wild-type M256 and mutant V256 residues. c: Random coil, e: Extended strand, h: Alpha helix, and t: Beta turn. **d** Prediction of an alpha-helix structure in the basic domain of bHLH25 and bHLH25^{M256V}. M256 is highlighted in red color, and V256 highlighted in cyan. **e**, **f** The replacement of bHLH25^{M256V} weakens the DNA binding ability of bHLH25 to bio-*pmiR397b*-G-box (**e**) and bio-*pCPS2*-N-box (**f**) in EMSA. GST-bHLH25 and GST-bHLH25^{M256V} pre-treated with H_2O_2 were purified with affinity beads separately, and were used in EMSA reaction free of H_2O_2. The red arrow indicates biotin labelled probes bound to GST-bHLH25 protein. **g-i** *bHLH25* mRNA levels (**g**), *miR397b* levels (**h**) and *CPS2* mRNA levels (**i**) were measured in three-week-old Kitaake, *bhlh25*-KO#6, and transgenic plants of *bHLH25* and *bHLH25^{M256V}* in *bhlh25*-KO#6 background. Data are mean ± s.d., n = 3 technical replicates; data were analyzed by one-way ANOVA with LSD test (**g-i**). Experiments were done with three biologically independent replications.
